# Trans-ethnic gut microbial signatures of prediabetic subjects from India and Denmark

**DOI:** 10.1186/s13073-021-00851-9

**Published:** 2021-03-03

**Authors:** Nishal Kumar Pinna, Ranjit Mohan Anjana, Shruti Saxena, Anirban Dutta, Visvanathan Gnanaprakash, Gnanavadivel Rameshkumar, Sukumaran Aswath, Srividhya Raghavan, Coimbatore Subramanian Shanthi Rani, Venkatesan Radha, Muthuswamy Balasubramanyam, Archana Pant, Trine Nielsen, Torben Jørgensen, Kristine Færch, Alireza Kashani, Maria Camila Alvarez Silva, Henrik Vestergaard, Tue Haldor Hansen, Torben Hansen, Manimozhiyan Arumugam, Gopinath Balakrish Nair, Bhabatosh Das, Oluf Pedersen, Viswanathan Mohan, Sharmila Shekhar Mande

**Affiliations:** 1 TCS Research, Tata Consultancy Services Limited, 54B Hadapsar Industrial Estate, Pune, 411013 India; 2 Madras Diabetes Research Foundation, No. 4, Conran Smith Road, Gopalapuram, Chennai, 600 086 India; 3 Molecular Genetics Laboratory, Infections and Immunology, Translational Health Science and Technology Institute, NCR Biotech Science Cluster, 3rd Milestone, Faridabad – Gurgaon Expressway, PO box #04, Faridabad, 121001 India; 4 Present address: SRM Medical College Hospital & Research Centre, SRM Institute of Science & Technology (SRMIST), Kattankulathur, Chennai, India; 5 Novo Nordisk Foundation Center for Basic Metabolic Research, University of Copenhagen, Blegdamsvej 3B, Maersk Tower, Building: 07-8-55, DK-2200 Copenhagen N, Denmark; 6 Center for Clinical Research and Prevention, Bispebjerg and Frederiksberg Hospitals, University of Copenhagen, Copenhagen, Denmark; 7 Steno Diabetes Center Copenhagen, Gentofte, Denmark; 8 Current affiliation: Qbiom, Microbiome Consultancy Service, Copenhagen, Denmark; 9 Department of Cardiology and Endocrinology, Slagelse Hospital, Slagelse, Denmark

## Abstract

**Background:**

Recent studies have indicated an association of gut microbiota and microbial metabolites with type 2 diabetes mellitus (T2D). However, large-scale investigation of the gut microbiota of “prediabetic” (PD) subjects has not been reported. Identifying robust gut microbiome signatures of prediabetes and characterizing early prediabetic stages is important for the understanding of disease development and could be crucial in early diagnosis and prevention.

**Methods:**

The current study performed amplification and sequencing on the variable regions (V1–V5) of the 16S rRNA genes to profile and compare gut microbiota of prediabetic individuals (*N* = 262) with normoglycemic individuals (*N* = 275) from two cohorts in India and Denmark. Similarly, fasting serum inflammatory biomarkers were profiled from the study participants.

**Results:**

After correcting for strong country-specific cohort effect, 16 operational taxonomic units (OTUs) including members from the genera *Prevotella9*, *Phascolarctobacterium*, *Barnesiella*, *Flavonifractor*, *Tyzzerella_4*, *Bacteroides*, *Faecalibacterium*, and *Agathobacter* were identified as enriched in normoglycaemic subjects with respect to the subjects with prediabetes using a negative binomial Wald test. We also identified 144 OTUs enriched in the prediabetic subjects, which included members from the genera *Megasphaera*, *Streptococcus*, *Prevotella9*, *Alistipes*, *Mitsuokella*, *Escherichia/Shigella*, *Prevotella2*, *Vibrio*, *Lactobacillus*, *Alloprevotella*, *Rhodococcus*, and *Klebsiella*. Comparative analyses of relative abundance of bacterial taxa revealed that the *Streptococcus*, *Escherichia/Shigella*, *Prevotella2*, *Vibrio*, and *Alloprevotella* OTUs exhibited more than fourfold enrichment in the gut microbiota of prediabetic subjects. When considering subjects from the two geographies separately, we were able to identify additional gut microbiome signatures of prediabetes. The study reports a probable association of *Megasphaera* OTU(s) with impaired glucose tolerance, which is significantly pronounced in Indian subjects. While the overall results confirm a state of proinflammation as early as in prediabetes, the Indian cohort exhibited a characteristic pattern of abundance of inflammatory markers indicating low-grade intestinal inflammation at an overall population level, irrespective of glycemic status.

**Conclusions:**

The results present trans-ethnic gut microbiome and inflammation signatures associated with prediabetes, in Indian and Danish populations. The identified associations may be explored further as potential early indicators for individuals at risk of dysglycemia.

**Supplementary Information:**

The online version contains supplementary material available at 10.1186/s13073-021-00851-9.

## Background

Type 2 diabetes mellitus (T2D) is a prevalent disease characterized by imbalances in regulation of blood glucose, and in the levels of blood lipids, blood platelet aggregation, and blood pressure [1–3]. Multiple gene variants associated with T2D have been identified, partly explaining the heritability of the disorder [4]. Apparently, the genetic susceptibility conferring risk of overt diabetes is triggered by numerous environmental risk factors including unhealthy diet, sedentary lifestyle, and smoking. Several of the diabetes-related environmental risk factors may mediate part of their diabetogenic impact through changes of the intestinal microbiota [5]. As such, aberrant composition and function of the intestinal microbiota have recently been implicated in the pathogenesis of T2D as well as several other metabolic disorders [6–11].

The T2D phenotype of Asian Indians is different from that of Europeans and is characterized by unique fat distribution, as well as changes in blood lipid composition and inflammatory markers [12, 13]. Several earlier studies have reported sub-clinical inflammation in the general Indian population in context of insulin resistance and prediabetes [12, 14–17]. Even when compared with other South Asian populations, it has been observed that while general adiposity could explain the difference in insulin resistance in Chinese and Malays, abdominal fat distribution and inflammation were the significant factors that contributed to excess insulin resistance in Asian Indians [17]. This characteristic phenotype of Asian Indians could possibly be linked to the gut microbiota through the unique dietary patterns of the Indian population. A distinctive feature of the gut microbiota of healthy Indian subjects is the predominance of the genera *Prevotella*, *Faecalibacterium*, *Collinsella*, and *Megasphaera* [18–21]. Furthermore, Asian Indians with T2D have been reported to have alterations in abundances of all kinds of microbes spanning Eubacteria, Archaea, and eukaryotes [22]. Elevated abundance of specific bacterial genera like *Escherichia* has also been reported in Indians with T2D when compared to healthy individuals [22, 23].

The natural history of T2D includes a stage of prediabetes (PD) where blood glucose levels are higher than normal, but not high enough to warrant the diagnosis of diabetes [24]. Prevention of disease progression is possible at this stage [25–28]. A few studies with limited sample size have reported a possible association between the gut microbiome composition and prediabetes [10, 29–33]. However, there have been no studies comparing different ethnicities, looking for a prediabetes signature in the gut microbiota. The current study aims to investigate the gut microbiota in Indian and Danish adults with normoglycemia and compare it with the microbiota of individuals with prediabetes in the two countries. Besides genetic differences between the Indian and Danish individuals, Denmark and India have entirely different cultural, climatic, socio-demographic and dietary patterns. This study is intended to serve as a unique resource in the quest to obtain specific microbiome signatures of prediabetes which can help in better understanding of the disease pathophysiology and may be explored further for identifying potential early indicators/ biomarkers for individuals with risk of dysglycemia, across populations of different ethnicities.

## Methods

### Participant enrollment and sample collection in Denmark

A total of 259 Danish volunteers [138 normoglycemic (NG) controls and 121 with prediabetes (PD)] were recruited from the DanFund [34] and ADDITION-PRO cohorts [35] and by advertisement in local newspapers. All Danish subjects were of White European ethnicity, aged 35 to 74 years, with a body mass index (BMI) from 20 to 40 kg/m^2^. Individuals with known diabetes of any kind, who were treated with antibiotics within 4 months, were pregnant or lactating, or unable to give informed consent were ineligible for inclusion.

Individuals with HbA1c below 5.7% (39 mmol/mol) and fasting plasma glucose below 6.1 mmol/L at time of screening were eligible for inclusion as normoglycemic controls. Individuals with a history of gestational diabetes were ineligible for inclusion as normoglycemic controls. Individuals with fasting plasma glucose of 6.1 to 6.9 mmol/L or glycated hemoglobin A1c of 5.7 to 6.4% (39 to 47 mmol/mol) were eligible for inclusion as prediabetics.

Fecal samples were collected by the participants following standardized procedures, including home sampling with immediate freezing at − 18 °C and transfer in an insulating polystyrene container with dry ice or cooling elements for final storage at − 80 °C within 48 h.

### Participant enrollment and sample collection in India

The Indian cohort comprised of 278 individuals [137 with normal glucose tolerance (NG) and 141with prediabetes (PD)] attending a tertiary care center for diabetes between April 2014 and April 2016. Diagnosis of normal glucose tolerance and impaired glucose tolerance was based on the results of a standard oral glucose tolerance test (OGTT), performed using a 82.5 g oral glucose load (equivalent to 75 g of anhydrous glucose). Study subjects were adults of either sex aged between 35 and 65 years. Individuals suffering from chronic and severe ailments (such as cancer and tuberculosis) and those who had used medications such as dipeptidyl peptidase-4 inhibitors, acarbose, glucagon-like peptide-1 receptor agonists, and orlistat were excluded from the study. A special kit containing the collection tubes, bed-pan liner, and dry ice required for collection of fecal samples were given to the study subjects. The fecal samples were frozen at − 20 °C within 1 h and then transferred to the − 80 °C freezer.

It may be noted here that although the current report pertains to microbial signatures associated with prediabetes (PD), the cohort recruitment in India and Denmark was done as part of a bigger research project “MicrobDiab - Studies of interactions between the gut Microbiome and the human host biology to elucidate novel aspects of the pathophysiology and pathogenesis of type 2 Diabetes”. The NG samples from India and Denmark reported in this work also forms the basis of a related study of the MicrobDiab project, aimed at deciphering the trans-ethnic microbial signatures associated with T2D.

### Phenotyping of study participants

Phenotyping of the study participants from both India and Denmark included recording basic physical variables, viz. height, weight, waist circumference, BMI, and blood pressure, along with a wide variety of biochemical tests and serum levels of 11 inflammation biomarkers (details in Additional file 1). In addition, a structured questionnaire was used to obtain information on age, gender, duration of prediabetes, family history of diabetes, food habits, physical activity patterns, smoking, allergic conditions, disease related to the gastrointestinal tract, etc.

### Microbiome sequencing

It may be noted here that to minimize confounding effects of the technical procedures, the standard operating procedures for recruitment of study participants, biological sample processing, and microbial DNA extraction of stools were synchronized. Furthermore, DNA sequencing of all samples were performed collectively in one sequencing center at the Translational Health Science and Technology Institute, India. Similarly, profiling of inflammation biomarkers from all samples were also performed in the same laboratory (details of protocols in Additional file 1).

Extraction of DNA was performed from 200 mg stool sample from each participant using a standard INRA protocol [36]. The variable regions (V1–V5) of the 16S rRNA genes were amplified using 27F(C1) and 926R(C5) primers followed by sequencing of the equimolar libraries performed on a 454 GS FLX+ pyrosequencer platform (Details in Supplementary Methods in Additional file 1). In addition to the samples collected from volunteers recruited in this study, 16S rRNA gene sequencing was also performed for additional microbiome samples collected from Indian and Danish volunteers with T2D for an allied study, using the same protocols and multiplexed sequencing runs mentioned above. Sequence data for all microbiome samples have been submitted to NCBI SRA and are available with SRA accession PRJNA517829 [37].

### Sequence analysis

The sequenced reads were demultiplexed using sequencing barcode information (Additional file 2: Table S1) and subsequently quality filtered (average PHRED score > 20). Considering a minimum sequencing coverage of 5000 high-quality reads per sample, a total of 18,380,379 sequences encompassing the V1–V5 region of the 16S rRNA gene were obtained from 864 microbiome samples. V3–V5 regions from all the sequenced reads (having variable read-lengths) were subsequently extracted using V-Xtractor 2.0 [38], and any read which did not encompass the complete V3–V5 region was not considered for further analysis. A total of 17,030,870 quality-checked and trimmed reads pertaining to 864 samples were considered for the downstream OTU picking step (average sequencing depth of 19,712 ± 7774 SD reads/sample). While many contemporary studies have preferred exact sequence variant-based analysis [39, 40] of amplicon sequencing data over OTU picking, these methods are mostly designed for processing of sequencing data generated on an Illumina platform, and to the best of our knowledge, there has been no validation of the utility of exact sequence variants vs OTUs on 454 single-end sequencing data. Further, resorting to identifying exact variants with 100% sequence identity may be construed as an attempt to go beyond what the accuracy of the sequencing technologies allows, and a more conservative OTU-based approach in context of noise arising from sequencing error, intra-genomic heterogeneity, etc., was preferred for the current study. OTU picking was performed using an “open reference OTU picking” approach as implemented in the QIIME pipeline v1.9.1 [41]. For the process, Greengenes OTUs clustered at 97% identity (Greengenes version 13_8) was used as the reference OTU database [42], while UCLUST v1.2.22q [43] was chosen as the preferred OTU picking method (“uclust_ref” run with default parameters for clustering sequences with 97% identity). Representative sequences from each of the OTUs were used for annotating corresponding taxonomic lineages (using the tool dada2 [39] considering SILVA database version 132 [44] as a reference). Sparse OTUs containing < 0.002% of the total number of high-quality reads sequenced were removed. A final OTU abundance table with a total of 1897 OTUs, including 1471 OTUs bearing correspondence to OTUs already cataloged in the Greengenes database, as well as 426 de novo OTUs identified from 864 samples was created. A subset of 537 microbiome samples pertaining to normoglycemic and prediabetic individuals from India and Denmark, corresponding to 10,647,149 quality-checked and trimmed reads, was considered for downstream taxonomic analyses in this study. Functional potential of the gut microbiomes were estimated from the taxonomic distribution using the tools PiCrust v1.1.0 [45] and Vikodak [46]. Although estimating functional potential of microbiomes should ideally be performed with appropriate shotgun metagenomics data, the current amplicon sequencing-based study has its limitation in this respect, and therefore used the above mentioned tools which are reported to provide reliable estimates from taxonomic abundance data.

### Statistical analysis

Alpha diversity metrics (viz. Shannon diversity, Simpson index and OTU richness) were calculated using R Vegan packagev2.5.2 [47]. Given that uneven sequencing depth of different samples may influence calculation of alpha diversity measures like OTU richness, this step was performed on rarefied abundance data (equivalent to the sample having minimum sequencing depth, i.e., ~ 4500 reads/sample). *T*-tests were performed to assess any significant differences between the alpha diversity parameters of samples belonging to different geographies or health status. Differences between the measured phenotypic traits of subjects belonging to different countries/health status were evaluated using Wilcoxon test(s). *P* values were corrected for multiple testing using Benjamini-Hochberg (BH) correction. PCoA plots based on taxonomic profiles (relative OTU abundance) of microbiome samples were generated the R Phyloseq package v1.22.3 [48], wherein weighted UniFrac was used as the distance metric. Similar PCoA plot was also generated using imputed functional profile of the microbiome samples (KEGG functional modules) wherein Jensen-Shannon divergence (JSD) was used as the distance metric. The extent of variation explained by geography and disease status was tested with permutational multivariate analysis of variance (PERMANOVA), using adonis2 function available in the R Vegan package v2.5.2. Dispersion of the country and disease status-specific clusters was evaluated using the *betadisper* function available in the R Vegan package v2.5.2. A negative binomial Wald test using the R package DESeq2v1.10.1 [49] was performed to identify the taxonomic groups (at all different levels of taxonomic hierarchy), which were differentially abundant in NG and PD samples (BH corrected *p* ≤ 0.05) for Indian and Danish cohorts separately. PD-specific microbiome abundance signatures were also evaluated after pooling together Indian and Danish cohorts, while correcting the negative binomial Wald test results for the anticipated geography-specific cohort effect. Further a forest-plot-based meta-analysis of the differentially abundant factors identified in the pooled analysis was also performed to put in context the effect sizes (log2 fold enrichment of mean abundances in PD with respect to NG) and directions in individual geographies. Additional negative binomial Wald tests were performed (using DESeq2) separately on Indian and Danish subjects to identify discriminating OTUs, while correcting for certain observed covariates of glycemic status, viz., waist-to-hip ratio, systolic BP, IL6, TNFα, LBP, and IAP, which might also influence the microbiome structure. Corrections were also performed for age and gender of the subjects, given that the age and gender distribution of normoglycemic and prediabetic cohorts from the two countries had some variations. Spearman correlations between abundances of differentially abundant microbial OTUs (between NG and PD subjects) and measured phenotypic traits of the subjects were calculated. It is relevant to mention here that HbA1c levels were used to define the NG and PD groups, and one might expect that correlations identified might be artifacts of the partitioning process. However, HbA1c levels for all subjects taken together were observed to follow a normal distribution (in both geographies), and therefore, partitioning of the subjects (NG/PD) based on clinically prescribed HbA1c thresholds is not expected to have any confounding effects on the computed correlations. Heatmaps depicting identified significant correlations were generated using the R “gplots” package v3.0.1. The correlations were evaluated separately for the Indian and Danish cohorts. Random forest (RF) classifier(s) were constructed for classifying PD samples based on gut microbiome composition using R Random forest package (v4.6–12). Detailed methods are provided in Additional file 1.

## Results

### Distinct phenotypes and inflammation marker levels in Indian and Danish cohorts

Table 1 (and Additional file 3: Table S2) shows the clinical and biochemical characteristics of the Danish individuals (normoglycemic = 138, prediabetic = 121) and Indian (normoglycemic = 137, prediabetic = 141) individuals participating in the study. Among Danish participants, individuals with prediabetes were significantly (Wilcoxon test, *p*_adj_ < 0.05) older, had higher waist to hip ratios, and higher systolic blood pressure compared to normoglycemic participants. While the clinical differences seen between the normoglycemic individuals and individuals with prediabetes in Denmark are as expected [50, 51], no significant differences in these respects could be observed in Indian participants with prediabetes when compared to the normoglycemic volunteers. When comparing countries, the Danish subjects with prediabetes were significantly older, taller, heavier, and had higher systolic blood pressure compared to their Indian counterparts. Similar differences in height, weight, and systolic blood pressure were also observed when normoglycemic individuals from both countries were compared. Another intriguing observation pertained to the magnitude of difference in HbA1c levels between the prediabetic and normoglycemic individuals from two countries. The normoglycemic individuals from India had an overall higher level of HbA1c (median = 37 mmol/mol) compared to the Danish normoglycemic participants (median = 33 mmol/mol). In effect, the difference between HbA1c levels of normoglycemic and prediabetic individuals appeared to be much higher in case of Danes, when compared to Indians.

**Table 1:** Differences in phenotypic traits in Danish and Indian cohorts

Parameter	Median (and IQR) value in Danish samples		Median (and IQR) value in Indian samples		Wilcoxon test ***p*** value (after BH correction)			
	NG	PD	NG	PD	NG vs PD (Denmark)	NG vs PD (India)	Denmark vs India (NG)	Denmark vs India (PD)
Age (years)	51(12)	**64(12)**	48(13)	51(14)	**2.18E−17**	2.7E**−**01	**4.04E−03**	**3.00E−20**
Gender	F = 90	F = 55	F = 74	F = 68	–	–	–	–
	M = 48	M = 66	M = 63	M = 73				
Height (cm)	170(15)	172(14)	159.4(14.7)	162.4(14.2)	9.50E**−**01	3.4E**−**01	**1.13E−16**	**2.74E−13**
Weight (kg)	79.1(15.3)	82.2(19.6)	67.3(21)	70(15.3)	7.79E**−**02	8.6E**−**02	**1.54E−09**	**3.50E−10**
Waist to hip ratio	0.89(0.135)	**0.94**(0.1)	0.89(0.11)	0.91(0.12)	**6.62E−06**	1.9E**−**01	3.09E**−**01	**9.18E−03**
BP systolic (mmHg)	128(23.8)	**137**(25)	120(24)	122(24)	**8.03E−04**	6.8E**−**01	**3.16E−04**	**4.53E−11**
BP diastolic (mmHg)	80(14)	83(15)	78(14)	79(12)	8.47E**−**02	5.5E**−**01	**2.22E−04**	**9.18E−03**
Glucose (mmol/L)	5.3(0.6)	**6.2**(0.5)	5(0.4)	**5.8**(0.6)	**9.17E−33**	**2.2E−27**	**1.22E−08**	**2.56E−14**
(mg/dL)	95.4(10.8)	**111.6**(9.0)	90(7.2)	**104.4**(10.8)				
HbA1c (DCCT%)	5.2(0.3)	**5.8**(0.2)	5.5(0.5)	**5.7**(0.6)	**9.50E−35**	**6.7E−11**	**2.06E−10**	6.61E**−**01
(mmol/mol)	33(3.3)	**40**(2.2)	37(5.5)	**39**(6.6)				
hsCRP (mg/L)	1.76(1.92)	2.08(2.84)	2.7(4.8)	2.8(5.1)	2.26E**−**01	5.6E**−**01	**7.09E−05**	**1.74E−03**
IL1β (pg/mL)	0.66(0.97)	0.87(0.74)	1.04(0.8)	1.07(0.9)	2.26E**−**01	5.6E**−**01	**2.61E−04**	**2.70E−03**
IL10 (pg/mL)	6.13(8.96)	5.18(7.54)	5.12(5.9)	5.19(5.8)	3.96E**−**01	7.3E**−**01	5.92E**−**02	8.80E**−**01
IL13 (pg/mL)	2.21(6.09)	2.70(6.07)	1.30(1.4)	1.39(2.0)	4.68E**−**01	5.6E**−**01	**8.54E−06**	**1.36E−04**
IL17A (pg/mL)	7.12(8.58)	5.90(6.23)	4.64(5.9)	5.09(5.7)	2.01E**−**01	5.6E**−**01	**8.50E−05**	1.55E**−**01
IL23 (pg/mL)	162.68(225.2)	190.40(174.6)	200.7(259.2)	213.3(290.1)	4.68E**−**01	9.7E**−**01	5.92E**−**02	3.40E**−**01
IL6 (pg/mL)	1.45(1.63)	**2.08**(3.16)	2.26(1.6)	2.32(2.1)	**4.67E−04**	5.6E**−**01	**2.45E−06**	2.46E**−**01
MCP1 (pg/mL)	1238.04(666.6)	1378.70(690)	838.8(846.1)	689.6(689.2)	2.26E**−**01	3.8E**−**01	**8.71E−09**	**6.27E−17**
TNFα (pg/mL)	2.80(1.66)	**3.66**(1.84)	5.15(3.3)	5.23(4.3)	**1.35E−05**	1.0E+ 00	**1.51E−19**	**3.50E−08**
LBP (μg/mL)	11.23(6.98)	**13.84**(7.56)	16.74(8.4)	15.4(8.1)	**7.93E−04**	5.6E**−**01	**3.29E−10**	**4.69E−02**
IAP (μg/mL)	0.32(0.21)	**0.37**(0.27)	0.35(0.32)	0.36(0.27)	**3.91E−02**	5.6E**−**01	2.97E**−**01	7.90E**−**01

Abbreviations: *BP* blood pressure, *HbA1c* glycated hemoglobin, *hsCRP* high-sensitive C-reactive protein, *IL1β* interleukin 1β, *IL10* interleukin 10, *IL13* interleukin 13, *IL17A* interleukin 17A, *IL23* interleukin 23, *IL6* interleukin 6, *MCP1* monocyte-chemoattractant protein 1, *TNFα* tumor necrosis factor α, *LBP* lipopolysachharide-binding protein, *IAP* intestinal alkaline phosphatase

Note: The significantly different parameters (Benjamini-Hochberg-corrected *p*_adj_ < 0.05) in the PD group from each cohort are highlighted in bold face fonts

Results on a panel of 11 fasting serum inflammatory biomarkers are also presented in Table 1. Among Danes, individuals with prediabetes had significantly (Wilcoxon test, *p*_adj_ < 0.05) higher levels of interleukin 6 (IL6), tumor necrosis factor α (TNFα), lipopolysaccharide-binding protein (LBP), and intestinal alkaline phosphatase (IAP) compared to normoglycemic individuals. In the Indian cohort, there were no significant differences in any of the circulating inflammatory markers in individuals with prediabetes when compared to those with normoglycemia. Interestingly, irrespective of the glycemic status, the overall levels of high-sensitivity C-reactive protein (hsCRP), TNFα, and LBP were significantly higher among Indians compared to Danes (Additional file 4: Table S3). On the other hand, the overall levels of interleukin 13 (IL13) and monocyte-chemoattractant protein 1 (MCP1) were higher among Danes. Considering the inter-individual variations in the biomarkers, we also reanalyzed the data from normoglycemic and prediabetic subjects while dividing into tertiles and found some interesting insights (Additional file 5: Table S4). Among Danes, while considering the tertile-based data analysis (particularly the tertile 2 and/or tertile 3 levels of biomarkers), most of the inflammatory markers were significantly higher in individuals with prediabetes compared to normoglycemic individuals. The only exceptions were interleukin 10 (IL10) and interleukin 17A (IL17A) levels, which were significantly lower in individuals with prediabetes. Similar analysis in Indians showed significantly higher levels of inflammatory biomarkers like high-sensitive C-reactive protein (hsCRP), IL1β, IL13, IL17A, IL6, TNFα, and IAP in individuals with prediabetes compared to normoglycemic individuals.

### Dominant and core bacterial taxa in Danish and Indian gut microbiota

While the gut microbiota of Danish participants were significantly (*t*-test, *p* < 0.05) more diverse when compared to the Indian volunteers (Additional file 6: Figure S1), no significant differences in alpha diversity were observed between microbiota belonging to the normoglycemic and prediabetic groups in the respective cohorts. Firmicutes, followed by Bacteroidetes, were the dominant phyla across all samples in both populations (Additional file 6: Figure S2, Additional file 7: Table S5A). While Actinobacteria, Proteobacteria, and Elusimicrobia were seen to be present in significantly (negative binomial Wald test; Benjamini-Hochberg-corrected *p*_adj_ < 0.05) higher proportions in the Indian cohort, Bacteroidetes, Tenericutes, Verrucomicrobia, and Synergistetes were observed to be significantly enriched in the Danish subjects. When resolved at a family level (Additional file 6: Figure S3, Additional file 7: Table S5B), Ruminococcaceae, Bacteroidaceae, Rikenellaceae, and Christensenellaceae were among the major families which exhibited more than twofold enrichment in Danes compared to Indians (negative binomial Wald test; Benjamini-Hochberg-corrected *p*_adj_ < 0.05). In contrast, Prevotellaceae, Veillonellaceae, Erysipelotrichaceae, Lactobacillaceae, Coriobacteriaceae, Streptococcaceae, and Atopobiaceae were seen to be significantly enriched (over twofold) in the Indian cohort.

**Fig. 1:**
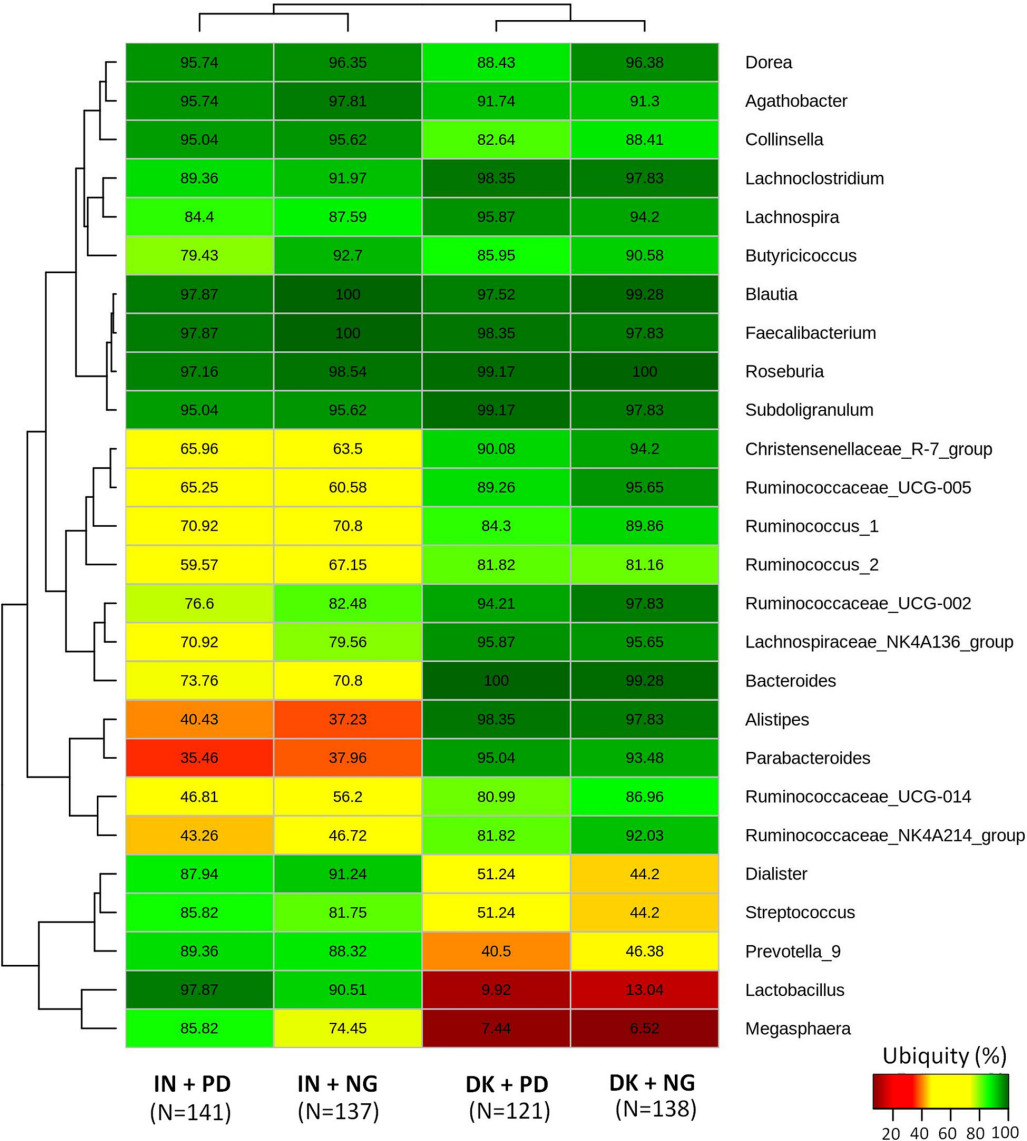
Core genera indifferent groups. Core genera identified in normoglycemic (NG) and prediabetic (PD) groups of samples corresponding to the Indian (IN) and Danish (DK) cohorts. Genera which are present in at least 80% of the samples belonging to a particular group, having a minimum (normalized) abundance of 0.1%, have been defined to constitute the core. The values indicated in the heatmap represent ubiquity of a taxon as a percentage of samples (in the respective groups) wherein the taxon is present at a relative abundance of ≥ 0.1%

A search for core genera (present in at least 80% of the subjects with minimum 0.1% abundance) in the gut microbiota of normoglycemic and prediabetic individuals showed *Dorea*, *Agathobacter*, *Collinsella*, *Lachnoclostridium*, *Lachnospira*, *Blautia*, *Faecalibacterium*, *Roseburia*, and *Subdoligranulum* to be present ubiquitously in subjects from both ethnic groups (Fig. 1). Although *Megasphaera* and *Lactobacillus* could be identified as a core microbiota in the gut of Indian subjects, their prevalence was very low in the Danish population. On the other hand, *Parabacteroides* and *Alistipes* were only present in a small fraction of the Indian samples, but could be identified as core genera in the Danish population. Strong geography-specific patterns were identified in the distribution of core OTUs (Additional file 8: Table S6). While a total of 32 OTUs were observed to be ubiquitously present across samples from both the geographies with normalized abundance> 0.01%, OTUs specific to the Danish (29 OTUs) and Indian (16 OTUs) participants could also be identified. Out of the 29 core OTUs specific to the Danish samples, 17 were Firmicutes, while 11 belonged to the phylum Bacteroidetes, including 6 from the genus *Bacteroides*. In contrast, the Indian cohort had only 3 Bacteroidetes OTUs, all from the genus *Prevotella9*, along with 12 Firmicutes OTUs and a single OTU belonging to the genus *Senegalimassilia* (phylum Actinobacteria).

### Gut microbiome composition in individuals with prediabetes

**Fig. 2:**
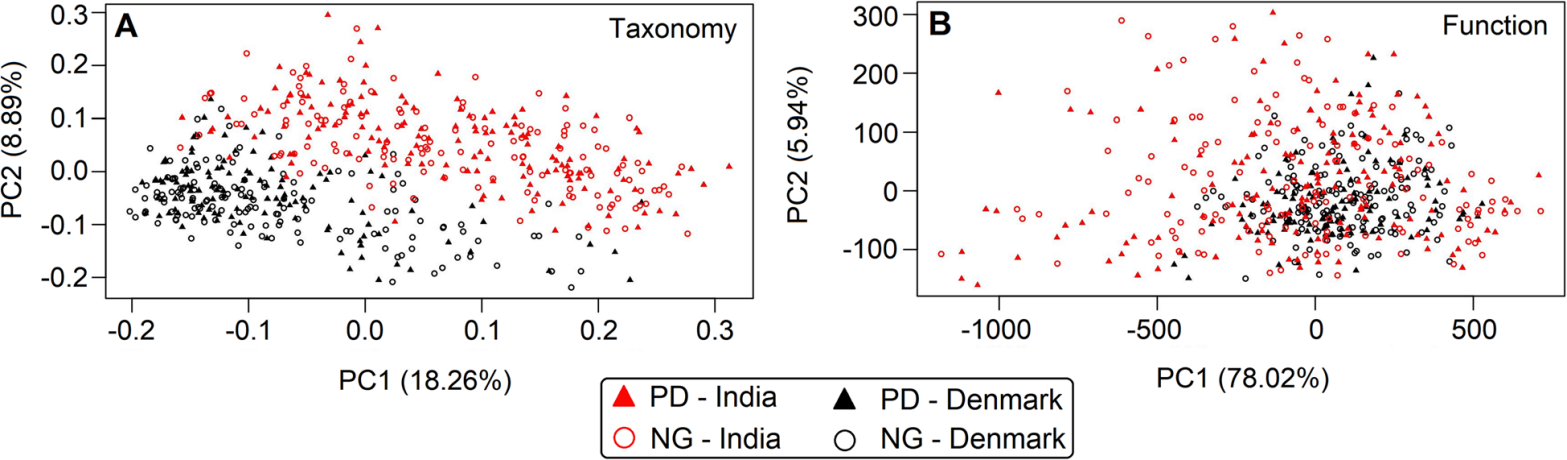
Taxonomic and functional diversity of microbiomes. PCoA plots based on **a** OTU presence using weighted Unifrac distances and **b** KEGG functional modules present in different microbiome samples (as inferred with Picrust) using JSD distances. The microbiome samples have been plotted along the first two principal components

Principal coordinate analysis (PCoA) based on OTU abundance using weighted UniFrac distance (see Supplementary Methods in Additional file 1) did not reveal any prediabetes-specific patterns when the Danish and Indian samples were combined (Fig. 2a). Instead, a strong country-specific effect on gut microbiota was apparent from the distinct clustering of Indian and Danish samples. The strong effect of geography on the gut microbiome was also confirmed by a PERMANOVA test (*R*^2^ = 11.2%; *p* = 0.001). A negative binomial Wald test, after correcting for the country-specific cohort effect, identified 160 OTUs, which were differentially abundant (*p*_adj_ < 0.05) in the samples based on glycemic status (Additional file 9: Table S7). OTUs belonging to *Prevotella9*, *Phascolarctobacteriumfaecium*, *Barnesiellaintestinihominis*, *Flavonifractorplautii*, *Tyzzerellanexilis*, *Bacteroidesnordii*, *Faecalibacterium*, and *Agathobacter* were among the OTUs that were enriched in normoglycemic subjects by two folds or more (Table 2). In addition, three OTUs from the family Ruminococcaceae, and one OTU each from the families Muribaculaceae and Christensenellaceae had more than twofold enriched abundance in normoglycemic subjects. In contrast, OTUs enriched by two folds or more in the subjects with prediabetes included those belonging to *Megasphaera*, *Streptococcus*, *Prevotella9*, *Alistipes*, *Mitsuokella*, *Escherichia/Shigella*, *Prevotella2*, *Vibrio cholerae*, *Lactobacillus*, *Alloprevotella*, *Rhodococcus*, *Klebsiella* and two more belonging to the family Ruminococcaceae. A meta-analysis of the differentially abundant factors presented in Table 2 is provided in Additional file 6: Figure S4. The forest plot depicts the effect sizes and directions of the factors in individual geographies, as well as the combined effect size. For almost all the OTUs identified through negative binomial Wald test on the pooled data (after correcting for the country-specific cohort effect), the effect direction of microbial association with dysglycemia was observed to be same in both geographies. However, effect sizes showed geography-specific trends and in many cases did not attain statistically significant values in one of the geographies. OTUs which showed different effect directions included those belonging to *Phascolarctobacteriumfecium*, *Tyzerella_4 nexiilis*, *Eschirichia/Shigella*, *Prevotella2*, *Alloprevotella*, and one de novo OTU belonging to *Lactobacillus*. In most of these cases, the effect was significantly strong in one of the geographies, which influenced the combined effect during pooled analysis. Further, for one of the OTUs belonging to *Falvonifractor plautii*, contrasting effects were observed during pooled (cohort-effect corrected) and meta-analyses, which can probably be attributed to differences in fitting its taxonomic abundance data to negative binomial distributions, once for the pooled data and subsequently for the geography-specific abundance data.

**Table 2:** Differentially abundant OTUs between NG and PD subjects from both Danish and Indian cohorts

OTU IDs	Taxonomic affiliation	Log2 fold change	Log2 FC standard error	BH-adjusted ***p*** value	Mean % abundance
**Enriched in NG**					
291725	*Prevotella_9*	− 1.7680	0.5088	8.88E−03	0.2918
556835	*Phascolarctobacterium faecium*	− 1.5371	0.4627	1.38E−02	0.2914
190975	*Barnesiella intestinihominis*	− 1.6235	0.4381	4.56E−03	0.0567
335550	*Flavonifractor plautii*	− 1.1188	0.3509	1.86E−02	0.0457
4315782	*[Family]Ruminococcaceae*	− 2.4732	0.7731	1.83E−02	0.0147
659361	*Tyzzerella_4 nexilis*	− 2.4165	0.8183	3.45E−02	0.0097
583656	*Bacteroides nordii*	− 1.7518	0.3759	1.97E−04	0.0092
583256	*Faecalibacterium*	− 1.7176	0.2714	5.54E−08	0.0087
211935	*Agathobacter*	− 1.0416	0.3203	1.66E−02	0.0086
177679	[Family]Muribaculaceae	− 1.4553	0.4535	1.79E−02	0.0048
819181	*Ruminococcaceae_UCG-002*	− 1.0428	0.3378	2.38E−02	0.0033
593008	*Christensenellaceae_R-7_group*	− 1.7182	0.5400	1.88E−02	0.0033
denovo32180	*Ruminococcaceae_UCG-010*	− 2.0100	0.7128	4.73E−02	0.0030
**Enriched in PD**					
817140	*Megasphaera elsdenii*	1.7989	0.3362	1.12E−05	1.1755
349024	*Streptococcus equinus/gallolyticus/infantarius/lutetiensis*	2.0231	0.3458	7.59E−07	0.8744
339221	*Prevotella_9*	1.4210	0.3933	6.20E−03	0.8176
357046	*Alistipes finegoldii/onderdonkii*	1.3202	0.3080	8.10E−04	0.4661
13811	*Mitsuokella*	1.3869	0.3688	4.05E−03	0.3890
1111294	*Escherichia/Shigella albertii/ boydii/coli/dysenteriae/fergusonii/flexneri/sonnei/vulneris*	2.0982	0.3131	6.45E−09	0.3829
264967	*Megasphaera*	1.9031	0.3591	1.21E−05	0.3539
566899	[Family]Ruminococcaceae	1.0812	0.3529	2.50E−02	0.3489
269937	*Prevotella_2*	2.5823	0.5627	2.47E−04	0.2158
345899	*Prevotella_9*	1.5634	0.4830	1.69E−02	0.1898
1767788	*Vibrio cholerae*	3.3938	0.6447	1.37E−05	0.1604
denovo49732	*Lactobacillus*	1.1079	0.3289	1.21E−02	0.1374
546557	*Alloprevotella*	2.5351	0.5821	6.27E−04	0.1229
278795	*Rhodococcus baikonurensis/ boritolerans/degradans/ erythropolis/globerulus/hoagii/ opacus/qingshengii/rhodochrous*	1.1766	0.2996	2.43E−03	0.1102
211191	[Family]Ruminococcaceae	1.0446	0.3314	1.99E−02	0.1033

Differentially abundant OTUs between NG and PD subjects, belonging to the Indian and Danish cohorts (pooled together), identified using a negative binomial Wald test (corrected for geography-specific cohort effect). A positive log2 fold change value indicates higher relative abundance of the OTU in PD subjects and vice versa. *P* values were adjusted for multiple testing using Benjamini-Hochberg correction. Up to top 15 OTUs (sorted according to mean abundance values) which are at least twofold enriched (*p*_adj_ < 0.05) either in the NG or the PD group are listed

When the Indian and Danish cohorts were considered separately, additional OTUs discriminating between the normoglycemic and prediabetic groups could be identified (Tables 3, 4, Additional file 10: Table S8, Additional file 11: Table S9). A total of 89 OTUs were found to be differentially abundant (*p*_adj_ < 0.05) in either the normoglycemic or the prediabetic group in Indian subjects (Additional file 10: Table S8A). In the Danish cohort, 56 OTUs were found to be differentially abundant (*p*_adj_ < 0.05) in either of these two groups (Additional file 11: Table S9A). Normoglycemic subjects from India were characterized by an overabundance (two folds or more) of multiple OTUs belonging to the *Prevotella9* group (which includes *Prevotella copri*), along with a few OTUs belonging to the family Ruminococcaceae, including the short-chain fatty acid (SCFA) producing *Faecalibacterium* [52] (Table 3). The Danish normoglycemic subjects also exhibited enriched abundance (two folds or more) of OTUs from the family Ruminococcaceae, along with a few OTUs from the genera *Phascolarctobacterium* and *Oscillibacter* and three OTUs belonging to *Prevotella9* (Table 4). Indian participants with prediabetes, on the other hand, were enriched in OTUs belonging to the genera *Lactobacillus*, *Megasphaera*, *Subdoligranulum*, *Escherichia/Shigella*, *Dialister*, *Vibrio*, *Streptococcus*, *Achromobacter*, and *Blautia*. Overall, an enrichment of Firmicutes OTUs was apparent in the Indian prediabetics. In Danish subjects with prediabetes, multiple OTUs belonging to the genus *Bacteroides* and family Lachnospiraceae were enriched. Interestingly, several fold enrichments of two OTUs belonging to *Prevotella2* group (which includes *Prevotella stercorea*) were identified in the Danish subjects with prediabetes. It may however be noted that most of the prominent cohort-specific microbial associations with glycemic status listed in Tables 3 and 4 did not follow a similar significant trend in the other cohort. In some cases, certain discriminating OTUs (e.g., those belonging to *Prevotella9* specific to the Indian cohort) were absent in the other cohort.

**Table 3:** Differentially abundant OTUs between NG and PD subjects (Indian cohort)

OTU IDs	Taxonomic affiliation	Log2 fold change	Log2 FC standard error	BH-adjusted ***p*** value (***p***_**adj**_)	Mean % abundance	Log2 FC in Denmark (and ***p***_**adj**_ value)
**Enriched in NG (India)**						
526358 *	*Faecalibacterium*	− 1.4023	0.3430	2.00E−03	0.0272	0.2549 (0.7247)
363017 *	*Ruminococcaceae_UCG-002*	− 1.2560	0.2843	5.70E−04	0.0254	− 0.5792 (0.1898)
211935 *	*Agathobacter*	− 1.4328	0.4420	2.65E−02	0.0142	− 0.4730 (0.6316)
583256 *	*Faecalibacterium*	− 2.8392	0.3956	1.27E−10	0.0126	− 0.4154 (0.6150)
321743 *	*Prevotella_9*	− 1.5612	0.4585	1.75E−02	0.0097	− 0.8695 (NA)
177679	[Family]Muribaculaceae	− 1.8582	0.5292	1.35E−02	0.0062	− 1.1579 (0.5283)
3910247 *	*Alloprevotella*	− 2.7503	0.7581	9.27E−03	0.0056	0.5692 (NA)
4295618	*Prevotella_9*	− 1.6808	0.5665	4.82E−02	0.0054	0.0614 (NA)
denovo143775	*Prevotella_9*	− 4.3633	1.1914	8.48E−03	0.0037	NA
denovo94756	*Prevotella_9*	− 3.6581	1.1732	3.53E−02	0.0028	NA
**Enriched in PD (India)**						
1121530 *	*Lactobacillus ruminis*	1.6953	0.2990	1.56E−06	2.8598	− 0.5500 (0.7993)
264967 *	*Megasphaera*	2.7006	0.4177	1.54E−08	0.6834	0.1234 (NA)
361811 *	*Subdoligranulum*	1.6443	0.2547	1.54E−08	0.5463	0.2621 (0.6316)
1111294 *	*Escherichia/Shigella albertii/boydii/coli/dysenteriae/fergusonii/flexneri/sonnei/vulneris*	3.0148	0.3638	4.12E−14	0.5294	− 0.1181 (0.9126)
661,229 *	*GKS98_freshwater_group*	1.9029	0.5708	2.04E−02	0.4334	− 0.5283 (NA)
333178 *	*Lactobacillus ruminis*	1.4600	0.3529	1.73E−03	0.3985	− 1.2126 (0.6758)
1105343 *	*Ruminococcaceae_UCG-013*	1.2424	0.3720	2.02E−02	0.3401	0.7922 (0.0164)
583746 *	*Dialister succinatiphilus*	1.3217	0.4431	4.71E−02	0.3357	− 1.9138 (0.3338)
1767788	*Vibrio cholerae*	4.6414	0.8994	2.20E−05	0.3053	2.1842 (0.1198)
denovo49732 *	*Lactobacillus*	2.2333	0.3784	4.27E−07	0.2630	− 0.7778 (0.7655)
292057	*Lactobacillus phage/reuteri/salivarius*	1.8088	0.5630	2.80E−02	0.2333	1.0336 (NA)
813217 *	*Klebsiella aerogenes/oxytoca/pneumoniae*	1.2564	0.3633	1.50E−02	0.1797	0.5921 (0.7735)
355307 *	*Subdoligranulum*	1.4338	0.2712	1.27E−05	0.1645	0.4640 (0.3236)
558264 *	*Achromobacter insolitus/xylosoxidans*	1.6829	0.4317	3.73E−03	0.1429	0.1816 (NA)
328283 *	*Streptococcus*	1.9115	0.4641	1.81E−03	0.1397	0.6262 (NA)

Differentially abundant OTUs between NG and PD subjects, belonging to the Indian cohort, identified using a negative binomial Wald test. A positive log2 fold change value indicates higher relative abundance of the OTU in PD subjects and vice versa. *P* values were adjusted for multiple testing using Benjamini-Hochberg correction. Up to top 15 OTUs (sorted according to mean abundance values) which are at least twofold enriched (*p*_adj_ < 0.05) either in the NG or the PD group are listed. The rightmost column depicts the log2 fold change (if any) for the same OTU in the Danish cohort with the respective *p*_adj_ values in brackets. “NA” in the rightmost column indicates absence (or limited abundance and/or ubiquity) of the OTU in the Danish cohort. OTUs marked with an asterisk (*) were also found to exhibit significant differential abundance between NG and PD groups in the Indian cohort after correcting for following factors—age, gender, waist-to-hip ratio, systolic BP, IL6, TNFα, LBP, and IAP. Further details in Additional file 13 Tables S8A and S8B

**Table 4:** Differentially abundant OTUs between NG and PD subjects (Danish cohort)

OTU IDs	Taxonomic affiliation	Log2 fold change	Log2 FC standard error	BH-adjusted ***p*** value (***p***_**adj**_)	Mean % abundance	Log2 FC in India (and ***p***_**adj**_ value)
**Enriched in NG (Denmark)**						
530653	*Prevotella_9*	− 2.1696	0.6476	2.72E−02	0.5973	0.8215 (0.1061)
556835	*Phascolarctobacterium faecium*	− 2.5626	0.5608	6.30E−04	0.5759	0.4750 (0.8399)
840914	*Prevotella_9*	− 3.0817	0.7375	2.77E−03	0.2892	0.9944 (0.2645)
569244 *	[Order]Mollicutes_RF39	− 2.2544	0.5219	1.84E−03	0.0900	0.7955 (0.4533)
366352	*Ruminococcus_1*	− 1.3403	0.3808	1.75E−02	0.0898	− 0.3344 (0.8399)
300855	*Family_XIII_AD3011_group*	− 1.0533	0.3375	4.91E−02	0.0185	0.0672 (0.9621)
denovo21348	*Prevotella_9*	− 3.3626	1.0755	4.91E−02	0.0093	0.1213 (NA)
denovo64437	*Oscillibacter*	− 1.6014	0.4896	3.37E−02	0.0092	1.2417 (0.1523)
denovo154693	*Ruminococcaceae_UCG-005*	− 2.0648	0.5084	3.84E−03	0.0067	0.3536 (NA)
190220	*Ruminococcaceae_UCG-002*	− 1.7534	0.5639	4.91E−02	0.0039	2.5112 (0.0841)
denovo56680 *	*Ruminococcaceae_NK4A214_group*	− 2.0885	0.6214	2.69E−02	0.0034	0.2107 (NA)
**Enriched in PD (Denmark)**						
535375	*Bacteroides fragilis/ovatus*	1.2882	0.3102	2.91E−03	0.4990	0.0167 (0.9890)
212481 *	*Lachnoclostridium*	1.4087	0.2772	8.84E−05	0.4144	0.5648 (0.2787)
269937	*Prevotella_2*	4.5234	1.2712	1.64E−02	0.2741	− 0.9062 (0.4702)
187623 *	*Bacteroides fragilis/xylanisolvens*	1.1188	0.3283	2.44E−02	0.2018	0.4457 (0.6316)
212359	*Lachnospiraceae_NK4A136_group*	1.0452	0.3360	4.91E−02	0.1308	− 0.4899 (0.4494)
332732	*Bacteroides intestinalis*	3.1027	0.5724	1.68E−05	0.1138	1.9399 (0.2202)
370361	[Family]Lachnospiraceae	2.1163	0.4551	4.71E−04	0.1056	1.3856 (0.1324)
361108 *	*Lachnoclostridium*	5.2401	0.8119	7.70E−08	0.0944	1.5206 (0.3246)
302,538	*Prevotella_2*	4.8436	1.3556	1.64E−02	0.0728	− 0.1564 (0.9519)
297045	*Ruminiclostridium_9*	2.1947	0.3231	1.55E−08	0.0578	0.0173 (0.9894)
326662	*Bacteroides*	4.2505	1.3187	3.82E−02	0.0475	− 0.5035 (0.8716)
564806	*Lachnoclostridium*	1.8245	0.5267	2.04E−02	0.0408	− 0.0955 (0.9760)
4449055	*Bacteroides*	3.4554	0.9515	1.48E−02	0.0405	0.9578 (0.5667)
549635	*Blautia*	2.8875	0.8503	2.48E−02	0.0363	− 1.7466 (0.4146)
422283	*Ruminiclostridium_9*	1.3861	0.3352	2.96E−03	0.0307	− 0.3696 (0.8889

Differentially abundant OTUs between NG and PD subjects, belonging to the Danish cohort, identified using a negative binomial Wald test. A positive log2 fold change value indicates higher relative abundance of the OTU in PD subjects and vice versa. *P* values were adjusted for multiple testing using Benjamini-Hochberg correction. Up to top 15 OTUs (sorted according to mean abundance values) which are at least twofold enriched (*p*_adj_ < 0.05) either in the NG or the PD group are listed. The rightmost column depicts the log2 fold change (if any) for the same OTU in the Indian cohort with the respective *p*_adj_ values in brackets. “NA” in the rightmost column indicates absence (or limited abundance and/or ubiquity) of the OTU in the Indian cohort. OTUs marked with an asterisk (*) were also found to exhibit significant differential abundance between NG and PD groups in the Danish cohort after correcting for following factors—age, gender, waist-to-hip ratio, systolic BP, IL6, TNFα, LBP, and IAP. Further details in Additional file 14 Tables S9A and S9B

It may also be noted that the above observations present an overall view of microbial associations that can either be directly related to the glycemic status, or any associated comorbidities, or other intrinsic/extrinsic host factors relevant to the studied cohorts. While for the Indian subjects, none of the measured physical/ biochemical parameters (other than glucose levels or HbA1c) or inflammation markers, as reported in Table 1, showed significant variations between the normoglycemic and prediabetic cohorts, the Danish subjects showed differences in multiple parameters including waist-to-hip ratio, systolic BP, IL6, TNFα, LBP, and IAP levels, as well as differences in age and gender distribution of the normoglycemic and prediabetic volunteers who could be recruited for the study. Given this observation, negative binomial Wald tests were repeated on the data from Indian and Danish cohorts, while correcting for the mentioned covariates (see Additional file 1). It was intriguing to note that post correcting for covariates, 129 differentially abundant OTUs (*p*_adj_ < 0.05) were identified to be associated with either the normoglycemic or the prediabetic groups belonging to the Indian cohort (Additional file 10: Table S8B). As expected for the Indian cohort, most of the differentially abundant OTUs (64 out of 89) between normoglycemic and prediabetic groups, identified prior to correcting for covariates, were still observed to be significant (*p*_adj_ < 0.05) discriminating factors. Out of 25 OTUs exhibiting differential abundance of two folds or more (depicted in Table 3), 19 OTUs were identified to be significantly discriminating even after correcting for the covariates. However, in case of the Danish cohort, the number of differentially abundant OTUs (*p*_adj_ < 0.05), in either the normoglycemic or the prediabetic group, decreased to 39 after correcting for the mentioned covariates (Additional file 11: Table S9B). Out of these, only 11 OTUs were in common with the earlier obtained list (Additional file 11: Table S9A) of differentially abundant OTUs between Danish normoglycemic and prediabetic groups. Out of 26 OTUs from the Danish cohort exhibiting differential abundance of two folds or more (depicted in Table 4), only 5 OTUs were identified to be significantly discriminating after correcting for the covariates. This set included one OTU belonging to the order Mollicutes and another belonging to the family Ruminococcaceae, which were enriched in the Danish normoglycemic subjects, as well as two OTUs belonging to the genus *Lachnoclostridium* and one OTU belonging to the genus *Bacteroides*, which were enriched in the Danish prediabetic subjects.

Differences in gut microbiomes pertaining to normoglycemic and prediabetic individuals were also apparent at higher taxonomic ranks (Additional file 12: Table S10). A negative binomial Wald test, after correcting for country-specific cohort effect, indicated that the families Enterobacteriaceae, Enterococcaceae, Vibrionaceae, and Burkholderiaceae, all from the phylum Proteobacteria; Streptococcaceae from phylum Firmicutes; and Nocardiaceae from phylum Actinobacteria had relatively higher abundances (Benjamini-Hochberg-corrected *p*_adj_ < 0.05) in the PD samples. However, at the phylum level, no significant variations could be observed.

### Inferred functional profiles of gut microbiome

Principal coordinate analysis (PCoA) of predicted functional profiles (KEGG functional modules) based on Jensen-Shannon distances (see Supplementary Methods in Additional file 1) did not reveal any prediabetes-specific signatures (Fig. 2b), which was in line with the results obtained using taxonomic profiles (Fig. 2a). Intriguingly, and in contrast with the taxonomy-based PCoA analysis, no country-specific separation was apparent. However, dispersion of predicted functional profiles pertaining to Indian gut microbiomes was significantly higher than that of the Danish functional profiles (Additional file 6: Figure S5). Such dispersion was not observed when taxonomic compositions of Danish and Indian gut microbiota were tested.

Certain predicted functional pathways and modules discriminating between the normoglycemic and prediabetic subjects could be identified using negative binomial Wald tests (Additional file 13: Table S11, Additional file 14: Table S12, Additional file 15: Table S13). However, the fold enrichments of these predicted pathways and modules, in either of the normoglycemic or prediabetic groups, were minimal in most cases (average log2 fold change = 0.06). The predicted pathways included tyrosine metabolism and ascorbate and aldarate metabolism, as well as multiple xenobiotic degradation pathways that were enriched in subjects with prediabetes (Additional file 13: Table S11). On the other hand, d-glutamine and d-glutamate metabolism exhibited an inverse trend and were depleted in prediabetic subjects.

Investigating the predicted functional profile at the module level led to further insights (Additional file 14: Table S12, Additional file 15: Table S13). Multiple modules pertaining to transport of sugars and phosphotransferase system (PTS) were enriched in the gut microbiome of individuals with prediabetes, which is in line with previous observations [6, 53]. In addition, several predicted functional modules pertaining to drug resistance and efflux pumps were observed to be enriched in the microbiome of prediabetic subjects, suggesting increased exposure to antibiotics or other xenobiotics. One of the interesting observations pertains to the metabolism of the neurotransmitter gamma-aminobutyric acid (GABA shunt and GABA biosynthesis functions), which was predicted to be enriched in prediabetic subjects after correcting for country-specific cohort effect. The enrichment was more prominent in the Indian cohort and assumes importance in context of previous studies indicating effects of GABA on the islet beta cells [54].

### Association between gut microbiota and clinical biomarkers

For both Indian and Danish cohorts, a relatively small proportion of OTUs enriched in the normoglycemic subjects exhibited correlations with clinical variables and inflammatory biomarkers (Additional file 6: Figure S6, Additional file 16: Table S14). In the Indian subjects, these OTUs were predominantly from the genus *Prevotella9* (4 OTUs), along with one OTU each from the genera *Faecalibacterium*, *Agathobacter*, *Alloprevotella*, and one OTU belonging to the family Muribaculaceae. All other OTUs exhibiting significant correlation(s) with one or more phenotypic variables were enriched in prediabetic samples. It was interesting to note that a considerable fraction of these OTUs (7 de novo OTUs out of 10) belonged to the genus *Megasphaera*, most of which exhibited significant positive correlations with fasting plasma glucose and HbA1c levels, and weak negative correlations with HDL cholesterol and inflammation markers like TNFα and LBP. Another intriguing observation pertains to two OTUs belonging to the family Burkholderiaceae including the one from the lymphoid tissue-resident commensal bacterial (LRC) genus *Achromobacter* and another from the GKS98 freshwater group, which showed significant positive correlations with inflammatory biomarkers like IL10 and IL17A. An OTU belonging to *Faecalibacterium* (OTU 319275) was also observed to be positively correlated with IL10 and IL6 levels in the Indian cohort, which is in line with previous reports suggesting anti-inflammatory and IL10 inducing roles of some *Faecalibacterium* strains [55, 56]. The heatmap corresponding to Danish subjects showed a small but coherent grouping of OTUs enriched in normoglycemic participants, which included four OTUs from the family Ruminococcaceae (including one OTU each from the genus *Ruminococcus1* and *Oscillibacter*), an OTU belonging to the genus *Phascolarctobacterium faecium* and another two OTUs belonging to the order MollicutesRF39 and family XIII AD3011 group from the order Clostridiales. Almost all OTUs depicted in this heatmap, which were associated with Danish prediabetic subjects, belonged to the order Clostridiales. A considerable number of these were from the family Lachnospiraceae followed by those from the family Ruminococcaceae, both these families being ubiquitously present in Danish gut microbiota. A couple of *Prevotella2* OTUs exhibiting modest negative correlations with HDL cholesterol also pertained to this prediabetes-associated group of OTUs identified in the Danish population.

### Microbiome signature-based classifiers for indicating predisposition to dysglycemia

**Fig. 3:**
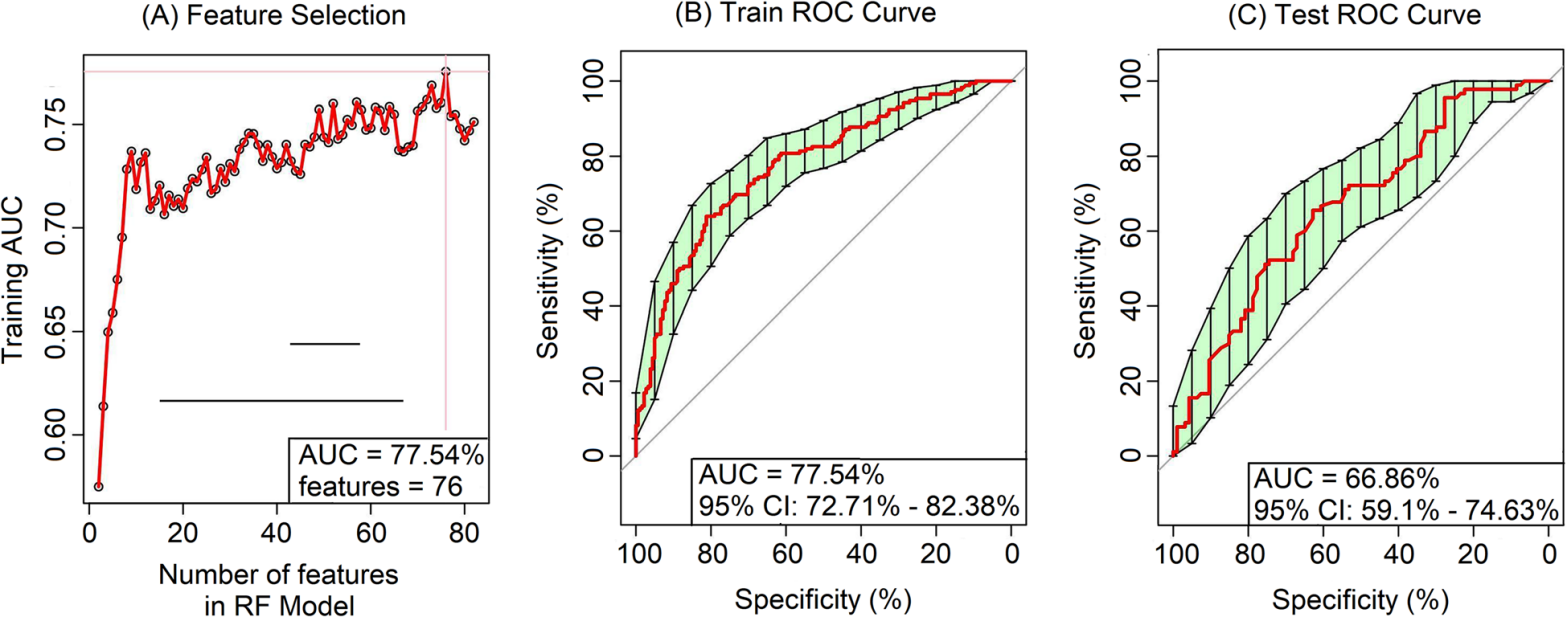
Microbiome-based random forest classifier for prediabetes. Performance of RF classifier (trained on OTU abundance) distinguishing between NG and PD samples from India and Denmark. [Note: The data was split into training and independent test sets (in the ratio of 66:34) and a feature selection step adopted while training the model performed with 10-fold cross-validation (× 10 replicates); Top 10 features were selected from each cross-validation fold and ranked based on their cumulative importance (gini score used). Final “bagged” RF model was built using a set of features providing best training AUC, selected through progressively adding the ranked features into the model (up to a maximum of 100), while evaluating training AUC]

Random forest (RF) classifiers were constructed to assess the ability of the abovementioned microbiome signatures in segregating the normoglycemic and prediabetic subjects (see Supplementary Methods in Additional file 1). When trained with taxonomic data (1897 OTUs as features) for all Indian and Danish gut microbiota samples, a RF model with area under the receiver operating characteristic curve (AUC) of 62.7% and out-of-bag (OOB) error rate of 40.04% could be obtained. However, the anticipated effect of extraneous predictors on a predictive model [57] and earlier reports of RF classifiers built on microbiome data [58], prompted adoption of an additional feature selection step. For feature selection, the whole dataset was randomly split (stratified considering proportions of NG and PD samples) into a training set and an independent test set in the ratio 66:34. Post feature selection step described in Additional file 1, a bagged RF model with 76 selected features (Additional file 17: Table S15) could be obtained with an improved AUC of 77.54%, and a decent test AUC of 66.86% (Fig. 3). While the clinical relevance of these RF models might be limited, results of this exercise reiterate the distinct gut microbiome signatures in prediabetic subjects from India and Denmark. Further, the set of selected OTUs (obtained through the feature selection step) used in the model holds relevance for future studies in this direction.

## Discussion

Recent evidences of causal or consequential relationship of the gut microbiota with metabolic phenotypes suggest the need of studying these aspects in each other’s context. The Danish and Indian cohorts were significantly different in multiple phenotypic aspects, and intriguingly the signs of metabolic syndrome like higher waist-to-hip ratios and systolic blood pressure were more apparent in the Danish prediabetic subjects. Similar patterns were also observed in levels of inflammatory biomarkers. While the Danish prediabetic subjects exhibited higher levels of several inflammatory biomarkers like IL6, TNFα, LBP, and IAP compared to normoglycemic individuals, there were no such differences between the Indian prediabetic and normoglycemic subjects. Notably, the fasting serum levels of a majority of inflammatory markers in Indian participants were higher than in the Danish participants. The only inflammatory markers having higher levels in the Danish participants included IL13 and MCP1, which have roles in allergic inflammation [59–61]. While several inflammatory markers have known association with T2D and the metabolic syndrome [62–66], an earlier study by Cappuccio and Miller [67] has also indicated ethnic differences in the level of circulating inflammatory markers which may be partially related to demographic, lifestyle, or genetic or gut microbiome factors. On the one hand, our observations suggest a state of proinflammation as early as in prediabetes. On the other hand, the observed characteristic pattern of inflammatory markers in the Indian cohort probably indicates prevalence of systemic and chronic intestinal inflammation at an overall population level. Higher levels of IL23, TNFα, and LBP have been reported to be associated with intestinal inflammation as well as systemic inflammation triggered by LPS and other bacterial products [68–70]. Recent studies also imply a role of IL-23/IL-17 pathway alterations in several disease states including T2D [71, 72] and our study supports the existence of these alterations as early as in prediabetes. In this context, the higher IAP values in Danish prediabetic subjects were in a subtle contrast with earlier reports on the role of IAP deficiency in metabolic syndrome [73], but this could reflect a mounting adaptive response to inflammation.

Comparing the Indian and Danish gut microbiota based on alpha diversity measures indicated higher diversity in the Danish cohort. This observation seems intriguing in context of earlier studies reporting higher alpha diversity of gut microbiota in many non-western populations [74–76]. However, reduced gut-microbial diversity is known to be associated with systemic inflammation [77, 78], and a relatively lower gut microbial diversity in the Indian subjects may be related to the observed levels of inflammatory biomarkers. Analyses investigating beta diversity, to some extent, echoed earlier findings pertaining to gut microbiota of Indian subjects, wherein the phylum Actinobacteria, and families Prevotellaceae, Veillonellaceae, and Streptococcaceae, were enriched, when compared to Americans [18]. In contrast, the Danish gut microbiota, profiled in the current study, was quite similar to that of the Americans and harbored a relatively larger proportion of microbes belonging to families like Ruminococcaceae, Bacteroidaceae, and Rikenellaceae. The observed distribution of core OTUs are also in line with our expectations pertaining to the characteristic features of Indian and Danish microbiota, such as a higher number of *Bacteroides* OTUs in the Danish samples and ubiquitous presence of *Prevotella* OTUs in the Indian samples [79–81]. The presence of a *Megasphaera* OTU in the Indian core set also concurs with observations made in recent Indian studies [18, 82].

Despite the strong country effect on the gut microbiota, certain taxonomic groups associated with prediabetes could be identified when the microbiome data from India and Denmark were pooled together. Additional taxonomic groups could also be identified when the microbiome data from the two countries were analyzed separately. Both the Danish and Indian normoglycemic subjects were enriched with multiple OTUs from the *Prevotella9* group as well as those belonging to the family Ruminococcaceae. A depletion of the butyrate producing family Ruminococcaceae has been reported earlier in Indian T2D subjects [22], as well as in Finnish prediabetic subjects [83]. On the other hand, the enrichment of OTUs belonging to pathogenic genera like *Vibrio* and *Streptococcus* in subjects with prediabetes was interesting, given the role of inflammation in diabetes. A recent study on Danish individuals with prediabetes has indicated significant enrichment of the genus *Streptococcus* and has suggested that the associated gut microbial alterations may be a signature of low-grade inflammation [30]. Enrichment of certain *Blautia* OTUs and depletion of bacteria belonging to Clostridialesvadin BB60 family noted in prediabetic subjects enrolled in the current study also appears to be coherent with the earlier observations pertaining to gut microbiota associated with Danish prediabetic subjects. However, the observation made in the earlier study pertaining to depletion of *Akkermansia muciniphila* in gut microbiota of Danish prediabetic individuals was not apparent in the current study population. An increased abundance of the genera *Lactobacillus* in prediabetic subjects, which was more prominent in the Indian population, could be correlated with earlier reports mentioning the genus’ association with T2D [22]. On the other hand, significant abundance of *Megasphaera* OTU(s) in Indian prediabetic subjects is a novel observation and particularly intriguing. Although *Megasphaera* has been reported to be a core gut microbe in the Indian population [18], its association with impaired glucose tolerance has not been reported earlier in any country or ethnicity. Multiple *Megasphaera* OTUs identified in the samples from Indian prediabetic subjects also exhibited significant positive correlations with fasting plasma glucose and HbA1c levels, and weak negative correlations with HDL cholesterol and inflammation markers like TNFα and LBP. Apart from a couple of recent studies on the Indian gut microbiome [18, 22], *Megasphaera* has not been reported to be a prevalent gut microbial taxon, especially in Caucasians. However, its role in lactate fermentation as well as its positive association with *Lactobacillus ruminis*, especially in cases of intestinal malabsorption, or increased availability of dietary sugars in the large intestine, has been reported [84]. The association of *Megasphaera* with Indian prediabetic subjects assumes importance in this context. Two other OTUs associated with Indian prediabetic subjects, belonging to the genus *Achromobacter* and GKS98 freshwater group (both belonging to the family Burkholderiaceae), showed significant positive correlations with the inflammation biomarkers IL10 and IL17A. Interestingly, many members of the family Burkholderiaceae, e.g., the genus *Achromobacter*, are known to constitute the group of lymphoid tissue-resident commensal (LRC) bacteria, which colonize the intestinal lymphoid tissue of healthy mammals [85]. The LRCs play a major role in intestinal immunity and are known to induce anti-inflammatory interleukins like IL10, IL6, IL1β, and IL17a. Another interesting observation pertained to enriched abundances of OTUs belonging to *Prevotella9* (which includes *Prevotella copri*) in both Indian and Danish normoglycemic subjects, and those belonging to *Prevotella2* (which includes *Prevotella stercorea*) in Danish prediabetic subjects. A couple of *Prevotella2* OTUs identified in the Danish population were also observed to exhibit modest negative correlations with HDL cholesterol. These observations probably reflect distinct roles of different *Prevotella* species in the gut and are in line with earlier findings indicating beneficial as well as pathogenic effects of members belonging to the genus *Prevotella* [86–88]. Microbiome composition is influenced by a multitude of factors, and while the current study did set out to find associations of microbial taxa with glycemic status, several of the measured covariates including physical/biochemical parameters of the subjects as well as the inflammation markers could have influenced the observed microbiome state. As discussed earlier, the phenotypic traits in normoglycemic and prediabetic subjects showed a deeper contrast in Danes than in Indians. Consequently, efforts towards identifying microbial association to dysglycemia in the Danish cohort, while correcting for the measured covariates using linear modeling, resulted in a fewer number of discriminating taxa between the normoglycemic and prediabetic gut microbiomes. In a sharp contrast though, correcting for covariates in the Indian cohort could fetch a higher number of discriminating taxonomic groups between the normoglycemic and prediabetic subjects. Literature suggests that microbiome signatures corresponding to different diseases and physiological conditions often overlap and can be a mixed effect from different host extrinsic and intrinsic factors [89]. The resultant microbiome shifts also are seldom unidirectional, with the microbiome sending feedback to the host, and in certain instances, modulating host factors. Given the limitations in identifying all possible underlying medical conditions as well as measuring all the potential confounders, confident assertions related to the disease-microbiome association (in this case with dysglycemia) remains difficult. Therefore, the lists of microbial taxa associated with the studied prediabetic and normoglycemic cohorts, both before and after correcting for the measured covariates, are presented in this report. It is likely that some of these observed associations, despite being statistically significant, may not be a direct outcome of glycemic status and may be related to associated comorbidities.

Functional potential of gut microbiomes inferred from 16S taxonomic profiles may not provide an estimate as accurate as that obtained with shotgun metagenomics or metatranscriptomics data. However, certain observations made in our study related to estimated enrichment of tyrosine metabolism, xenobiotic degradation, and ascorbate and aldarate metabolism in gut microbiota associated with prediabetes could be placed in context of earlier observations related to dysglycemia. Higher tyrosine levels have been associated with the risk of T2D [90]. A previous study has highlighted a higher proportion of bacterial genes related to xenobiotic degradation pathways harbored by the gut microbiome of Chinese subjects with T2D [6]. Gut bacteria of leptin-deficient transgenic mice with metabolic syndrome have been reported to show enrichment of ascorbate and aldarate metabolism [91]. Another interesting insight pertained to the inferred depletion of d-glutamine and d-glutamate metabolism, and enrichment of GABA metabolism functional modules in prediabetic gut microbiota. Previous studies in mice have indicated potential protective and regenerative effects of GABA on the islet beta cells [54], as well as the role of microbiota in modulating GABA and glutamate circuits [92]. Another study reported relatively higher GABA levels in subjects with T2D, and its possible impact on cognitive abilities [93]. Our observations hint at a probable association of the gut microbiota and GABA level modulation in early prediabetic stages. However, understanding the effects of this modulation with respect to insulin production or a progression to diabetic neuropathy requires further research.

The above observations, coupled with the results pertaining to phenotypic data as well as levels of inflammatory biomarkers, indicate that the role of gut microbiome in the pathophysiology of prediabetes in Indian subjects is different compared to that in Europeans. While chronic systemic inflammation appears to be characteristic of the Indian population in general, the observed anti-inflammatory and protective effects induced by various factors in the Indian gut microbiome appear to play key roles in defining gut-health status and modulating the onset and progression of diabetes.

## Conclusions

In complex metabolic disorders, identifying biological signatures at the onset of disease is crucial to reduce or prevent the rapid progression of disease. The compositional and functional potential alterations of gut microbiota and proinflammation observed in prediabetic subjects in the present study is an important and significant advancement. In fact, the importance of sub-clinical detection of gut microbial biomarkers of obesity and T2D has recently been emphasized by several others [94]. Microbial abundance patterns and distinct levels of inflammatory markers identified in this study appear as robust sub-clinical signatures of prediabetes and may be explored further as potential early indicators for individuals at risk of dysglycemia.

## Supplementary Information

**Additional file 1:** Supplementary methods. The file provides details of supplementary methods.

**Additional file 2:** Table S1. Details of 864 microbiome samples sequenced.Details of 864 microbiome samples sequenced indicating sequencing primers, barcodes and basic metadata.

**Additional file 3:** Table S2. Biochemical parameters measured in Danish and Indian cohorts. Comparison of additional biochemical parameters measured in Danish and Indian cohorts.

**Additional file 4:** Table S3. Country effect on level of inflammatory biomarkers. Country effect on level of inflammatory biomarkers estimated as differences (%) in means (IN relative to DK) derived from linear mixed models adjusted for HbA1c values. Significant effects after BH correction (p_adj_ < 0.05) are indicated with asterisks.

**Additional file 5:** Table S4. Differentially abundant inflammation markers between Indian NGT and PD samples.Table S4A: Differentially abundant inflammation markers between Indian NGT and PD samples identified using Wilcoxon test (for different tertile ranges). Differences significant at p_adj_ < 0.05 (Benjamini Hochberg corrected) are highlighted in the table. (Median and Interquartile ranges in NGT and PD groups have been indicated). Table S4B: Differentially abundant inflammation markers between Danish NGT and PD samples identified using Wilcoxon test (for different tertile ranges). Differences significant at p_adj_ < 0.05 (Benjamini Hochberg corrected) are highlighted in the table. (Median and Interquartile ranges in NGT and PD groups have been indicated).

**Additional file 6:** Figures S1-S6. PDF file with all supplementary figures (Figures S1-S6) with corresponding figure legends.

**Additional file 7: **Table S5. Differentially abundant phyla and families between Indian and Danish samples. Table S5A: Differentially abundant phyla between Indian (IN) and Danish (DK) gut microbiomes identified using a negative binomial Wald test. A positive log2 fold change value indicates higher relative abundance of the OTU in DK subjects and vice-versa. *P*-values were adjusted for multiple testing using Benjamini-Hochberg correction (p_adj_ < 0.05 are highlighted in bold). Phyla are sorted according their mean abundance (%) across datasets. Table S5B: Differentially abundant family between Indian (IN) and Danish (DK) gut microbiomes identified using a negative binomial Wald test. A positive log2 fold change value indicates higher relative abundance of the OTU in DK subjects and vice-versa. *P*-values were adjusted for multiple testing using Benjamini-Hochberg correction. Results significant at p_adj_ < 0.05 are depicted. More than two fold change in abundance have been highlighted in bold. Families are sorted according their mean abundance (%) across datasets.

**Additional file 8:** Table S6. Core OTUs identified in Indian and Danish cohorts (ignoring disease status). OTUs which are present in at least 80% of the samples belonging to a particular cohort, having a minimum (normalized) abundance of 0.01%, have been defined to constitute the core.

**Additional file 9: **Table S7. Differentially abundant OTUs between NG and PD subjects. Differentially abundant OTUs between NG and PD subjects, belonging to the Indian and Danish cohorts (pooled together) identified using a negative binomial Wald test (corrected for geography specific cohort effect). A positive log2 fold change value indicates higher relative abundance of the OTU in PD subjects and vice-versa. *P*-values were adjusted for multiple testing using Benjamini-Hochberg method. Significantly (p_adj_ < 0.05) discriminating OTUs are listed in Table.

**Additional file 10: **Table S8. Differentially abundant OTUs between NG and PD subjects of Indian cohort. Table S8A: Differentially abundant OTUs between NG and PD subjects, belonging to the Indian cohort, identified using a negative binomial Wald test. A positive log2 fold change value indicates higher relative abundance of the OTU in PD subjects and vice-versa. P-values were adjusted for multiple testing using Benjamini-Hochberg correction. Significantly (p_adj_ < 0.05) discriminating OTUs are listed in Table. The negative bionomial Wald test results for the same OTUs from the Danish cohort are also indicated for ease in comparison. ‘NA’ in the rightmost column indicate absence (or limited abundance) of the OTU in the Danish cohort. The complete Taxonomic Lineage of each OTU is also provided in rightmost columns. Table S8B: Differentially abundant OTUs between NG and PD subjects, belonging to the Indian cohort, identified after correcting for covariates (age, gender, waist-to-hip ratio, Systolic BP, IL6, TNFα, LBP and IAP) using a negative binomial Wald test. A positive log2 fold change value indicates higher relative abundance of the OTU in PD subjects and vice-versa. *P*-values were adjusted for multiple testing using Benjamini-Hochberg correction. Significantly (p_adj_ < 0.05) discriminating OTUs are listed in Table. The negative bionomial Wald test results for the same OTUs from the Danish cohort are also indicated for ease in comparison. ‘NA’ in the rightmost column indicate absence (or limited abundance) of the OTU in the Danish cohort. The complete Taxonomic Lineage of each OTU is also provided in rightmost columns.

**Additional file 11: **Table S9. Differentially abundant OTUs between NG and PD subjects of Danish cohort. Table S9A: Differentially abundant OTUs between NG and PD subjects, belonging to the Danish cohort, identified using a negative binomial Wald test. A positive log2 fold change value indicates higher relative abundance of the OTU in PD subjects and vice-versa. *P*-values were adjusted for multiple testing using Benjamini-Hochberg correction. Significantly (p_adj_ < 0.05) discriminating OTUs are listed in Table. The negative bionomial Wald test results for the same OTUs from the Indian cohort are also indicated for ease in comparison. ‘NA’ in the rightmost column indicate absence (or limited abundance) of the OTU in the Indian cohort. The complete Taxonomic Lineage of each OTU is also provided in rightmost columns. Table S9B: Differentially abundant OTUs between NG and PD subjects, belonging to the Danish cohort, identified after correcting for covariates (age, gender, waist-to-hip ratio, Systolic BP, IL6, TNFα, LBP and IAP) using a negative binomial Wald test. A positive log2 fold change value indicates higher relative abundance of the OTU in PD subjects and vice-versa. *P*-values were adjusted for multiple testing using Benjamini-Hochberg correction. Significantly (p_adj_ < 0.05) discriminating OTUs are listed in Table. The negative bionomial Wald test results for the same OTUs from the Indian cohort are also indicated for ease in comparison. ‘NA’ in the rightmost column indicate absence (or limited abundance) of the OTU in the Indian cohort. The complete Taxonomic Lineage of each OTU is also provided in rightmost columns.

**Additional file 12: **Table S10. Differentially abundant phyla, family and genera between NG and PD subjects. Table S10A: Differentially abundant phyla between NG and PD subjects, belonging to the Indian and Danish cohorts (pooled together), identified using a negative binomial Wald test (corrected for geography specific cohort-effect). A positive log2 fold change value indicates higher relative abundance of the OTU in PD subjects and vice-versa. *P*-values were adjusted for multiple testing using Benjamini-Hochberg correction. Table S10B: Differentially abundant family between NG and PD subjects, belonging to the Indian and Danish cohorts (pooled together), identified using a negative binomial Wald test (corrected for geography specific cohort-effect). A positive log2 fold change value indicates higher relative abundance of the OTU in PD subjects and vice-versa. *P*-values were adjusted for multiple testing using Benjamini-Hochberg correction. Significantly (p_adj_ < 0.05) discriminating families are listed in Table. Table S10C: Differentially abundant genera between NG and PD subjects, belonging to the Indian and Danish cohorts (pooled together), identified using a negative binomial Wald test (corrected for geography specific cohort-effect). A positive log2 fold change value indicates higher relative abundance of the OTU in PD subjects and vice-versa. P-values were adjusted for multiple testing using Benjamini-Hochberg correction. Significantly (p_adj_ < 0.05) discriminating genera are listed in Table.

**Additional file 13:** Table S11. Differentially abundant KEGG pathways (level 3) between NG and PD subjects. Differentially abundant KEGG pathways (level 3) between NG and PD subjects, belonging to the Indian and Danish cohorts (pooled together), identified using a negative binomial Wald test (corrected for geography specific cohort-effect). A positive log2 fold change value indicates higher relative abundance of the Pathway in PD subjects and vice-versa. P-values were adjusted for multiple testing using Benjamini-Hochberg correction (list of pathways sorted according to p_adj_values). Pathways that are differentially abundant at a significance level of p_adj_ < 0.05 are listed.

**Additional file 14:** Table S12. Differentially abundant KEGG functional modules between NG and PD subjects. Differentially abundant KEGG functional modules between NG and PD subjects, belonging to the Indian and Danish cohorts (pooled together), identified using a negative binomial Wald test (corrected for geography specific cohort-effect). A positive log2 fold change value indicates higher relative abundance of the module in PD subjects and vice-versa. P-values were adjusted for multiple testing using Benjamini-Hochberg method (list of modules sorted according to p_adj_ values). Top 15 differentially abundant modules are listed.

**Additional file 15:** Table S13. Differentially abundant KEGG functional modules between NG and PD subjects belonging to (S13A) Denmark and (S13B) India. Differentially abundant KEGG functional modules between NG and PD subjects belonging to (S13A) Denmark and (S13B) India, identified using negative binomial Wald tests. A positive log2 fold change value indicates higher relative abundance of the module in PD subjects in the respective geography and vice-versa. P-values were adjusted for multiple testing using Benjamini-Hochberg method (list of modules sorted according to p_adj_values). Modules that are differentially abundant at a significance level of p_adj_ < 0.05 are listed.

**Additional file 16: **Table S14. Correlations between Discriminating OTUs and phenotypic traits includingbiochemical and inflammatory markers. Table S14A: Spearman correlations between discriminating OTUs and phenotypic traits including biochemical and inflammatory markers in the Indian cohort. Correlation values between each discriminating OTU against different biochemical and inflammatory markers were also corrected for multiple testing using Benjamini-Hochberg (BH) correction. *P* < 0.05 are listed and p_adj_ ≤ 0.05 are highlighted in red. Table S14B: Spearman correlations between discriminating OTUs and phenotypic traits including biochemical and inflammatory markers in the Danishcohort.Correlation values between eachdiscriminating OTU against different biochemical and inflammatory markers were also corrected for multiple testing using Benjamini-Hochberg (BH) correction. P < 0.05 are listed and p_adj_ ≤ 0.05 are highlighted in red.

**Additional file 17:** Table S15. Features selected for Random Forest classifier. 76 OTUs obtained during feature selection step while building random forest classifier for identifying PD samples. Features (OTUs), along with their Gini importance score and effect on performance of RF model in terms of ‘mean decrease in accuracy’ during cross validation step is provided.

## Data Availability

Sequence data for all microbiome samples has been submitted to NCBI SRA and are available with SRA accession PRJNA517829 (https://www.ncbi.nlm.nih.gov/bioproject/PRJNA517829/) [37].

## Abbreviations

NGT: Normal glucose tolerance
PD: Prediabetes
HbA1c: Glycated hemoglobin
IL6: Interleukin 6
TNFα: Tumor necrosis factor α
LBP: Lipopolysachharide-binding protein
IAP: Intestinal alkaline phosphatase
IL13: Interleukin 13
MCP1: Monocyte-chemoattractant protein 1
IL10: Interleukin 10
IL17A: Interleukin 17A
hsCRP: High-sensitive C-reactive protein
IL1β: Interleukin 1β
IAP: Intestinal alkaline phosphatase
SCFA: Short-chain fatty acid
*p*_adj_: Adjusted *p* value
PCoA: Principal coordinate analysis

## References

[1] Sami W, Ansari T, Butt NS, Hamid MRA (2017). Effect of diet on type 2 diabetes mellitus: a review. Int J Health Sci (Qassim).

[2] Wu Y, Ding Y, Tanaka Y, Zhang W (2014). Risk factors contributing to type 2 diabetes and recent advances in the treatment and prevention. Int J Med Sci.

[3] Stumvoll M, Goldstein BJ, van Haeften TW (2005). Type 2 diabetes: principles of pathogenesis and therapy. Lancet..

[4] Prasad RB, Groop L (2015). Genetics of type 2 diabetes-pitfalls and possibilities. Genes (Basel).

[5] Lynch SV, Pedersen O (2016). The human intestinal microbiome in health and disease. N Engl J Med.

[6] Qin J, Li Y, Cai Z, Li S, Zhu J, Zhang F (2012). A metagenome-wide association study of gut microbiota in type 2 diabetes. Nature..

[7] Larsen N, Vogensen FK, van den Berg FWJ, Nielsen DS, Andreasen AS, Pedersen BK (2010). Gut microbiota in human adults with type 2 diabetes differs from non-diabetic adults. PLoS One.

[8] Sanz Y, Olivares M, Moya-Pérez Á, Agostoni C (2015). Understanding the role of gut microbiome in metabolic disease risk. Pediatr Res.

[9] Okubo H, Nakatsu Y, Kushiyama A, Yamamotoya T, Matsunaga Y, Inoue M-K (2018). Gut microbiota as a therapeutic target for metabolic disorders. Curr Med Chem.

[10] Zhang X, Shen D, Fang Z, Jie Z, Qiu X, Zhang C (2013). Human gut microbiota changes reveal the progression of glucose intolerance. PLoS One.

[11] Aydin Ö, Nieuwdorp M, Gerdes V. The gut microbiome as a target for the treatment of type 2 diabetes. Curr Diab Rep. 2018;18 [cited 2018 Sep 25]. Available from: https://www.ncbi.nlm.nih.gov/pmc/articles/PMC6013535/10.1007/s11892-018-1020-6 PMC6013535 29931613

[12] Gujral UP, Pradeepa R, Weber MB, Narayan KV, Mohan V (2013). Type 2 diabetes in south Asians: similarities and differences with white Caucasian and other populations. Ann N Y Acad Sci.

[13] Unnikrishnan R, Anjana RM, Mohan V (2014). Diabetes in South Asians: is the phenotype different?. Diabetes..

[14] Chambers JC, Eda S, Bassett P, Karim Y, Thompson SG, Gallimore JR (2001). C-reactive protein, insulin resistance, central obesity, and coronary heart disease risk in Indian Asians from the United Kingdom compared with European whites. Circulation..

[15] Chandalia M, Cabo-Chan AV, Devaraj S, Jialal I, Grundy SM, Abate N (2003). Elevated plasma high-sensitivity C-reactive protein concentrations in Asian Indians living in the United States. J Clin Endocrinol Metab.

[16] Gokulakrishnan K, Mohanavalli KT, Monickaraj F, Mohan V, Balasubramanyam M (2009). Subclinical inflammation/oxidation as revealed by altered gene expression profiles in subjects with impaired glucose tolerance and type 2 diabetes patients. Mol Cell Biochem.

[17] Gao H, Salim A, Lee J, Tai ES, van Dam RM (2012). Can body fat distribution, adiponectin levels and inflammation explain differences in insulin resistance between ethnic Chinese, Malays and Asian Indians?. Int J Obes.

[18] Bhute S, Pande P, Shetty SA, Shelar R, Mane S, Kumbhare SV (2016). Molecular characterization and meta-analysis of gut microbial communities illustrate enrichment of Prevotella and Megasphaera in Indian subjects. Front Microbiol.

[19] Das B, Ghosh TS, Kedia S, Rampal R, Saxena S, Bag S (2018). Analysis of the gut microbiome of rural and urban healthy Indians living in sea level and high altitude areas. Sci Rep.

[20] Tandon D, Haque MM, Saravanan R, Shaikh S, Sriram P, Dubey AK (2018). A snapshot of gut microbiota of an adult urban population from Western region of India. PLoS One.

[21] Chakravarthy SK, Jayasudha R, Ranjith K, Dutta A, Pinna NK, Mande SS (2018). Alterations in the gut bacterial microbiome in fungal keratitis patients. PLoS One.

[22] Bhute SS, Suryavanshi MV, Joshi SM, Yajnik CS, Shouche YS, Ghaskadbi SS (2017). Gut microbial diversity assessment of Indian type-2-diabetics reveals alterations in Eubacteria, Archaea, and eukaryotes. Front Microbiol.

[23] Pushpanathan P, Srikanth P, Seshadri KG, Selvarajan S, Pitani RS, Kumar TD (2016). Gut microbiota in type 2 diabetes individuals and correlation with monocyte chemoattractant protein1 and interferon gamma from patients attending a tertiary care centre in Chennai, India. Indian J Endocrinol Metab.

[24] Faerch K, Vaag A, Holst JJ, Hansen T, Jørgensen T, Borch-Johnsen K (2009). Natural history of insulin sensitivity and insulin secretion in the progression from normal glucose tolerance to impaired fasting glycemia and impaired glucose tolerance: the Inter99 study. Diabetes Care.

[25] Nah E-H, Chu J, Kim S, Cho S, Kwon E. Efficacy of lifestyle interventions in the reversion to normoglycemia in Korean prediabetics: one-year results from a randomised controlled trial. Prim Care Diabetes. 2018;13:212–20. 10.1016/j.pcd.2018.11.017 30583933

[26] Perreault L, Pan Q, Mather KJ, Watson KE, Hamman RF, Kahn SE (2012). Effect of regression from prediabetes to normal glucose regulation on long-term reduction in diabetes risk: results from the Diabetes Prevention Program Outcomes Study. Lancet..

[27] Knowler WC, Barrett-Connor E, Fowler SE, Hamman RF, Lachin JM, Walker EA (2002). Reduction in the incidence of type 2 diabetes with lifestyle intervention or metformin. N Engl J Med.

[28] Weber MB, Ranjani H, Staimez LR, Anjana RM, Ali MK, Narayan KMV (2016). The stepwise approach to diabetes prevention: results from the D-CLIP randomized controlled trial. Diabetes Care.

[29] Ciubotaru I, Green SJ, Kukreja S, Barengolts E (2015). Significant differences in fecal microbiota are associated with various stages of glucose tolerance in African American male veterans. Transl Res.

[30] Allin KH, Tremaroli V, Caesar R, Jensen BAH, Damgaard MTF, Bahl MI (2018). Aberrant intestinal microbiota in individuals with prediabetes. Diabetologia..

[31] Lambeth SM, Carson T, Lowe J, Ramaraj T, Leff JW, Luo L (2015). Composition, diversity and abundance of gut microbiome in prediabetes and type 2 diabetes. J Diabetes Obes.

[32] Egshatyan L, Kashtanova D, Popenko A, Tkacheva O, Tyakht A, Alexeev D (2016). Gut microbiota and diet in patients with different glucose tolerance. Endocr Connect.

[33] Crusell MKW, Hansen TH, Nielsen T, Allin KH, Rühlemann MC, Damm P (2018). Gestational diabetes is associated with change in the gut microbiota composition in third trimester of pregnancy and postpartum. Microbiome..

[34] Dantoft TM, Ebstrup JF, Linneberg A, Skovbjerg S, Madsen AL, Mehlsen J (2017). Cohort description: the Danish study of functional disorders. Clin Epidemiol.

[35] Johansen NB, Hansen A-LS, Jensen TM, Philipsen A, Rasmussen SS, Jørgensen ME (2012). Protocol for ADDITION-PRO: a longitudinal cohort study of the cardiovascular experience of individuals at high risk for diabetes recruited from Danish primary care. BMC Public Health.

[36] Godon JJ, Zumstein E, Dabert P, Habouzit F, Moletta R (1997). Molecular microbial diversity of an anaerobic digestor as determined by small-subunit rDNA sequence analysis. Appl Environ Microbiol.

[37] MicrobDiab consortium. MicrobDiab - Studies of interactions between the gut microbiome and the human host biology to elucidate novel aspects of the pathophysiology and pathogenesis of type 2 Diabetes. Bioproject PRJNA517829. NCBI SRA. https://www.ncbi.nlm.nih.gov/bioproject/PRJNA517829/ (2019).

[38] Hartmann M, Howes CG, Abarenkov K, Mohn WW, Nilsson RH (2010). V-Xtractor: an open-source, high-throughput software tool to identify and extract hypervariable regions of small subunit (16S/18S) ribosomal RNA gene sequences. J Microbiol Methods.

[39] Callahan BJ, McMurdie PJ, Rosen MJ, Han AW, Johnson AJA, Holmes SP (2016). DADA2: high-resolution sample inference from Illumina amplicon data. Nat Methods.

[40] Amir A, McDonald D, Navas-Molina JA, Kopylova E, Morton JT, Zech Xu Z, et al. Deblur rapidly resolves single-nucleotide community sequence patterns. mSystems. 2017;2:e00191–16. 10.1128/mSystems.00191-16 PMC5340863 28289731

[41] Caporaso JG, Kuczynski J, Stombaugh J, Bittinger K, Bushman FD, Costello EK (2010). QIIME allows analysis of high-throughput community sequencing data. Nat Methods.

[42] DeSantis TZ, Hugenholtz P, Larsen N, Rojas M, Brodie EL, Keller K (2006). Greengenes, a chimera-checked 16S rRNA gene database and workbench compatible with ARB. Appl Environ Microbiol.

[43] Edgar RC (2010). Search and clustering orders of magnitude faster than BLAST. Bioinformatics..

[44] Pruesse E, Quast C, Knittel K, Fuchs BM, Ludwig W, Peplies J (2007). SILVA: a comprehensive online resource for quality checked and aligned ribosomal RNA sequence data compatible with ARB. Nucleic Acids Res.

[45] Langille MGI, Zaneveld J, Caporaso JG, McDonald D, Knights D, Reyes JA (2013). Predictive functional profiling of microbial communities using 16S rRNA marker gene sequences. Nat Biotechnol.

[46] Nagpal S, Haque MM, Mande SS (2016). Vikodak--a modular framework for inferring functional potential of microbial communities from 16S metagenomic datasets. PLoS One.

[47] Oksanen J, Blanchet FG, Kindt R, Legendre P, Minchin PR, O’hara RB (2013). Package ‘vegan.’ Community ecology package, version.

[48] McMurdie PJ, Holmes S (2013). phyloseq: an R package for reproducible interactive analysis and graphics of microbiome census data. PLoS One.

[49] Love MI, Huber W, Anders S (2014). Moderated estimation of fold change and dispersion for RNA-seq data with DESeq2. Genome Biol.

[50] Meisinger C, Rückert IM, Stöckl D, Thorand B, Peters A, Kowall B (2014). Hematological parameters and prediabetes and diabetes in adults from the general population: a cross-sectional study. J Diabetes Metab.

[51] Tabák AG, Herder C, Rathmann W, Brunner EJ, Kivimäki M (2012). Prediabetes: a high-risk state for developing diabetes. Lancet..

[52] Anand S, Kaur H, Mande SS (2016). Comparative in silico analysis of butyrate production pathways in gut commensals and pathogens. Front Microbiol.

[53] Manor O, Borenstein E (2017). Revised computational metagenomic processing uncovers hidden and biologically meaningful functional variation in the human microbiome. Microbiome..

[54] Soltani N, Qiu H, Aleksic M, Glinka Y, Zhao F, Liu R (2011). GABA exerts protective and regenerative effects on islet beta cells and reverses diabetes. Proc Natl Acad Sci U S A.

[55] Rossi O, van Berkel LA, Chain F, Tanweer Khan M, Taverne N, Sokol H (2016). Faecalibacteriumprausnitzii A2-165 has a high capacity to induce IL-10 in human and murine dendritic cells and modulates T cell responses. Sci Rep.

[56] Sokol H, Pigneur B, Watterlot L, Lakhdari O, Bermúdez-Humarán LG, Gratadoux J-J (2008). Faecalibacteriumprausnitzii is an anti-inflammatory commensal bacterium identified by gut microbiota analysis of Crohn disease patients. Proc Natl Acad Sci U S A.

[57] Kuhn M, Johnson K, Johnson K. Feature engineering and selection: a practical approach for predictive models [Internet]: Chapman and Hall/CRC; 2019. [cited 2020 Jan 16]. Available from: https://www.taylorfrancis.com/books/9781315108230

[58] Feng Q, Liang S, Jia H, Stadlmayr A, Tang L, Lan Z (2015). Gut microbiome development along the colorectal adenoma-carcinoma sequence. Nat Commun.

[59] de Vries JE (1998). The role of IL-13 and its receptor in allergy and inflammatory responses. J Allergy Clin Immunol.

[60] Deshmane SL, Kremlev S, Amini S, Sawaya BE (2009). Monocyte chemoattractant protein-1 (MCP-1): an overview. J Interf Cytokine Res.

[61] Ip WK, Wong CK, Lam CWK (2006). Interleukin (IL)-4 and IL-13 up-regulate monocyte chemoattractant protein-1 expression in human bronchial epithelial cells: involvement of p38 mitogen-activated protein kinase, extracellular signal-regulated kinase 1/2 and Janus kinase-2 but not c-Jun NH2-terminal kinase 1/2 signalling pathways. Clin Exp Immunol.

[62] Dinarello CA, Donath MY, Mandrup-Poulsen T (2010). Role of IL-1beta in type 2 diabetes. CurrOpin Endocrinol Diabetes Obes.

[63] Harder-Lauridsen NM, Krogh-Madsen R, Holst JJ, Plomgaard P, Leick L, Pedersen BK (2014). Effect of IL-6 on the insulin sensitivity in patients with type 2 diabetes. Am J Physiol Endocrinol Metab.

[64] Mirza S, Hossain M, Mathews C, Martinez P, Pino P, Gay JL (2012). Type 2-diabetes is associated with elevated levels of TNF-alpha, IL-6 and adiponectin and low levels of leptin in a population of Mexican Americans: a cross-sectional study. Cytokine..

[65] Moreno-Navarrete JM, Ortega F, Serino M, Luche E, Waget A, Pardo G (2012). Circulating lipopolysaccharide-binding protein (LBP) as a marker of obesity-related insulin resistance. Int J Obes (Lond).

[66] Rodríguez-Hernández H, Simental-Mendía LE, Rodríguez-Ramírez G, Reyes-Romero MA (2013). Obesity and inflammation: epidemiology, risk factors, and markers of inflammation. Int J Endocrinol.

[67] Miller MA, Cappuccio FP (2007). Ethnicity and inflammatory pathways - implications for vascular disease, vascular risk and therapeutic intervention. Curr Med Chem.

[68] Ahern PP, Izcue A, Maloy KJ, Powrie F (2008). The interleukin-23 axis in intestinal inflammation. Immunol Rev.

[69] van der Bruggen T, Nijenhuis S, van Raaij E, Verhoef J, van Asbeck BS (1999). Lipopolysaccharide-induced tumor necrosis factor alpha production by human monocytes involves the raf-1/MEK1-MEK2/ERK1-ERK2 pathway. Infect Immun.

[70] Sakura T, Morioka T, Shioi A, Kakutani Y, Miki Y, Yamazaki Y (2017). Lipopolysaccharide-binding protein is associated with arterial stiffness in patients with type 2 diabetes: a cross-sectional study. Cardiovasc Diabetol.

[71] Fatima N, Faisal SM, Zubair S, Siddiqui SS, Moin S, Owais M (2017). Emerging role of interleukins IL-23/IL-17 axis and biochemical markers in the pathogenesis of type 2 diabetes: association with age and gender in human subjects. Int J Biol Macromol.

[72] Babaie F, Hasankhani M, Mohammadi H, Safarzadeh E, Rezaiemanesh A, Salimi R (2018). The role of gut microbiota and IL-23/IL-17 pathway in ankylosing spondylitis immunopathogenesis: new insights and updates. Immunol Lett.

[73] Bilski J, Mazur-Bialy A, Wojcik D, Zahradnik-Bilska J, Brzozowski B, Magierowski M (2017). The role of intestinal alkaline phosphatase in inflammatory disorders of gastrointestinal tract. Mediat Inflamm.

[74] Yatsunenko T, Rey FE, Manary MJ, Trehan I, Dominguez-Bello MG, Contreras M (2012). Human gut microbiome viewed across age and geography. Nature..

[75] Martínez I, Stegen JC, Maldonado-Gómez MX, Eren AM, Siba PM, Greenhill AR (2015). The gut microbiota of rural Papua New Guineans: composition, diversity patterns, and ecological processes. Cell Rep.

[76] De Filippo C, Cavalieri D, Di Paola M, Ramazzotti M, Poullet JB, Massart S (2010). Impact of diet in shaping gut microbiota revealed by a comparative study in children from Europe and rural Africa. Proc Natl Acad Sci U S A.

[77] Guinane CM, Cotter PD (2013). Role of the gut microbiota in health and chronic gastrointestinal disease: understanding a hidden metabolic organ. Ther Adv Gastroenterol.

[78] Nishikawa J, Kudo T, Sakata S, Benno Y, Sugiyama T (2009). Diversity of mucosa-associated microbiota in active and inactive ulcerative colitis. Scand J Gastroenterol.

[79] Dehingia M, Devi KT, Talukdar NC, Talukdar R, Reddy N, Mande SS (2015). Gut bacterial diversity of the tribes of India and comparison with the worldwide data. Sci Rep.

[80] Gupta VK, Paul S, Dutta C (2017). Geography, ethnicity or subsistence-specific variations in human microbiome composition and diversity. Front Microbiol.

[81] Deschasaux M, Bouter KE, Prodan A, Levin E, Groen AK, Herrema H (2018). Depicting the composition of gut microbiota in a population with varied ethnic origins but shared geography. Nat Med.

[82] Kumbhare SV, Kumar H, Chowdhury SP, Dhotre DP, Endo A, Mättö J (2017). A cross-sectional comparative study of gut bacterial community of Indian and Finnish children. Sci Rep.

[83] Org E, Blum Y, Kasela S, Mehrabian M, Kuusisto J, Kangas AJ (2017). Relationships between gut microbiota, plasma metabolites, and metabolic syndrome traits in the METSIM cohort. Genome Biol.

[84] Patrone V, Vajana E, Minuti A, Callegari ML, Federico A, Loguercio C (2016). Postoperative changes in fecal bacterial communities and fermentation products in obese patients undergoing bilio-intestinal bypass. Front Microbiol.

[85] Fung TC, Bessman NJ, Hepworth MR, Kumar N, Shibata N, Kobuley D (2016). Lymphoid-tissue-resident commensal bacteria promote members of the IL-10 cytokine family to establish mutualism. Immunity..

[86] Kovatcheva-Datchary P, Nilsson A, Akrami R, Lee YS, De Vadder F, Arora T (2015). Dietary fiber-induced improvement in glucose metabolism is associated with increased abundance of Prevotella. Cell Metab.

[87] Ley RE (2016). Gut microbiota in 2015: Prevotella in the gut: choose carefully. Nat Rev Gastroenterol Hepatol.

[88] Pedersen HK, Gudmundsdottir V, Nielsen HB, Hyotylainen T, Nielsen T, Jensen BAH (2016). Human gut microbes impact host serum metabolome and insulin sensitivity. Nature..

[89] Schmidt TSB, Raes J, Bork P (2018). The human gut microbiome: from association to modulation. Cell..

[90] de Mello VD, Paananen J, Lindström J, Lankinen MA, Shi L, Kuusisto J (2017). Indolepropionic acid and novel lipid metabolites are associated with a lower risk of type 2 diabetes in the Finnish Diabetes Prevention Study. Sci Rep.

[91] Connor SC, Hansen MK, Corner A, Smith RF, Ryan TE (2010). Integration of metabolomics and transcriptomics data to aid biomarker discovery in type 2 diabetes. Mol BioSyst.

[92] Mazzoli R, Pessione E (2016). The neuro-endocrinological role of microbial glutamate and GABA signaling. Front Microbiol.

[93] van Bussel FCG, Backes WH, Hofman PAM, Puts NAJ, Edden RAE, van Boxtel MPJ (2016). Increased GABA concentrations in type 2 diabetes mellitus are related to lower cognitive functioning. Medicine (Baltimore).

[94] Yassour M, Lim MY, Yun HS, Tickle TL, Sung J, Song Y-M (2016). Sub-clinical detection of gut microbial biomarkers of obesity and type 2 diabetes. Genome Med.

