# Supplementary material for: Trans-ethnic gut microbial signatures of prediabetic subjects from India and Denmark
Source: Genome Med. 2021 Mar 3;13:36. doi: 10.1186/s13073-021-00851-9 (PMC7931552; doi:10.1186/s13073-021-00851-9)
Supplement: Supplementary file 1 — Additional file 1: Supplementary methods. The file provides details of supplementary methods. [file 13073_2021_851_MOESM1_ESM.docx]

**Additional File 1: Supplementary Methods**

**Ethical committee approval**

The Indian substudy received appropriate ethics approvals from the Institutional Ethics Committee of the Madras Diabetes Research Foundation (MDRF/NCT/09-06/2013).

The Danish substudy was approved by the Ethical Committees of the Capital Region of Denmark (H-3-2013-102) and the Danish Data Protection Agency (2013-54-0498) and conducted in accordance with the Helsinki declaration.

Informed consent was obtained from all volunteers in Indian and Denmark prior to inclusion.

**Study design and sample collection**

Danish sub-study

259 Danish volunteers, 138 normoglycaemic controls and 121 with prediabetes, were recruited from the DanFund [1] and ADDITION-PRO cohorts [2] and by advertisement in local newspapers. All Danish subjects were of White European ethnicity, aged 35 to 74 years, with a body-mass index from 20 to 40 kg/m^2^. Individuals with known diabetes of any kind, who were treated with antibiotics within 4 months, were pregnant or lactating, or unable to give informed consent were ineligible for inclusion.

Individuals with a glycated haemoglobin A1c below 5.7% (39 mmol/mol) and fasting plasma glucose below 6.1 mmol/L at time of screening were eligible for inclusion as normoglycaemic controls. Individuals with a history of gestational diabetes were ineligible for inclusion as normoglycaemic controls.

Individuals with fasting plasma glucose of 6.1 to 6.9 mmol/L or glycated haemoglobin A1c of 5.7 to 6.4% (39 to 47 mmol/mol) were eligible for inclusion as prediabetics.

Volunteers were examined in the morning following a 10-h overnight fast. Participants were weighed on an electronic scale (TANITA BC-420MA, Tanita Corporation of America, USA) without shoes, dressed in light indoor clothing or underwear after having emptied their bladder. Height was measured to the nearest 0.5 cm without shoes, using a wall-mounted stadiometer (ADE MZ10023, ADE, Hamburg, Germany). Body-mass index (BMI) was calculated as weight in kg divided by the square of height in meters. Waist and hip circumference were measured to the nearest 1 cm in erect position midway between the iliac crest and the lower costal margin, and at the level of the pubic symphysis, respectively. Body composition was assessed using bioelectric impedance analysis (TANITA BC-420MA, USA). Blood pressure was recorded as the mean of duplicate measurements on the non-dominant arm, in reclined position after a 5-min rest.

Blood was collected by puncture of the antecubital vein. Plasma glucose was analysed by the glucose oxidase method using a colorimetric slide test on a Vitros 5600 system (Ortho Clinical Diagnostics, USA; CV 6.1%). Plasma triglyceride (TG), total cholesterol (TC), and high-density lipoprotein (HDL) were analysed on a Vitros 5600 system (CV 14.6%, 11.6%, and 17.0%, respectively). Very-low-density lipoprotein (VLDL) was calculated as VLDL=0.45×TG. Low-density lipoprotein was calculated as LDL=TC−HDL−VLDL. Haemoglobin A1c (HbA1c) was analysed by high-performance liquid chromatography (HPLC) on a TOSOH G8 system (Tosoh Bioscience, San Francisco, CA USA, CV 7.2%).

Faecal samples were collected by the participants following standardized procedures, including home sampling with immediate freezing at ‒18°C and transfer in an insulating polystyrene container with dry ice or cooling elements for final storage at -80°C within 48 h.

Indian sub-study

The Indian cohort comprised 278consecutive individuals [137with normal glucose tolerance (NGT) and 141with prediabetes (PD)] attending a tertiary care centre for diabetes between April 2014 and April 2016 (month and year of recruitment). Diagnosis of NGT and IGT was based on the results of a standard oral glucose tolerance test (OGTT), performed using an 82.5 g oral glucose load (equivalent to 75 g of anhydrous glucose). Study subjects were adults of either gender aged between 35 to 65 years. Individuals suffering from chronic and severe ailments (such as cancer and tuberculosis) and those who had used medications such as dipeptidyl peptidase-4 inhibitors, acarbose, glucagon-like peptide-1 receptor agonists and orlistat, were excluded from the study.

A structured questionnaire was used to obtain information on age, gender, duration of prediabetes, family history of diabetes, food habits, physical activity patterns, smoking, allergic conditions, disease related to the gastrointestinal tract etc.

Weight, height and waist circumference were obtained by trained data collectors using standardized methods. BMI was calculated as weight (kg) divided by height (m) squared. Blood pressure was recorded from the right arm in a sitting position to the nearest 2 mmHg with a mercury sphygmomanometer (Diamond Deluxe BP apparatus, Pune, India). Two readings were taken 5 min apart, and the mean of the two was taken as the blood pressure. From all participants fasting blood samples were collected for measurement of a wide variety of biochemical tests. A special kit containing the collection tubes, bedpan liner and dry ice required for collection of fecal samples were given to the study subjects. The fecal samples were frozen at -20°C within one hour and then transferred to the -80°C freezer.

All biochemical assays including measurement of Fasting plasma glucose (hexokinase method), serum cholesterol (cholesterol oxidase– peroxidase–amidopyrine method), serum triglycerides (glycerol phosphate oxidase–peroxidase–amidopyrine method) and HDL cholesterol (direct method–polyethylene glycol–pretreated enzymes) were measured using Hitachi- 912 Autoanalyzer (Hitachi, Mannheim, Germany). Low density lipoprotein (LDL) cholesterol was calculated using the Friedewald formula. Glycated hemoglobin (HbA1c) was measured by high-performance liquid chromatography using the Variant machine (Bio-Rad, Hercules, California, USA). Participants with normal glucose tolerance (NGT) and impaired glucose tolerance (IGT) were diagnosed using oral glucose tolerance test (OGTT) as per the World Health Organization (WHO) Consulting Criteria. NGT was defined if the 2-h post-glucose value was < 7.8mmol/L (140mg/dL) and FPG was < 6.1 mmol/L (100 mg/dL). Individuals with IGT was diagnosed if the 2-h post-glucose value was >7.8 mmol/L (140mg/dL) and < 11.1 mmol/L (200mg/ dL). All measurements were performed in the laboratory at the study site which is certified by the College of American Pathologists (Northfield, IL) (No. 7214031) and the National Accreditation Board for Testing and Calibration of Laboratories (New Delhi, India) (M0226).

Glucose tolerance status was determined from a standard 75g 2 hour glucose tolerance test in the healthy control subjects and in prediabetes and estimates of insulin secretion and sensitivity were calculated. Subjects with a 2 hour plasma glucose value between 7.8 mmol/L (140mg/dl) and 11.1 mmol/L (200 mg/dl) were classified as IGT or Prediabetes and those with a 2hr plasma glucose value <7.8 mmol/L (140mg/dl) as NGT. All biochemical assays including measurement of fasting and post prandial plasma glucose and lipids were carried out using a corning Express Plus Auto Analyser (Corning, Medfield, MA, USA) using Boehringer Mannheim kits. Glycosylated haemolglobin (HbA1c) was estimated by HPLC using the variant machine (Bio-Rad, Hercules, CA, USA), while C-peptide concentrations were measured using DAKO C- peptide ELISA kit (Dako, Denmark).

It may be noted here that to minimize confounding effects of the technical procedures, the standard operating procedures for recruitment of study participants, biological sample processing and microbial DNA extraction of stools were synchronized. Furthermore, DNA sequencing of all samples were performed collectively in one sequencing center. Similarly, profiling of inflammation biomarkers from all samples were also performed in the same laboratory and details are given below.

**Estimation of Metabolite Biomarkers**

A panel of serum cytokines (IL10, IL13, IL17A, IL1β, IL23, IL6, and TNFα) were measured by custom multiplex immunoassay (Human High Sensitivity T Cell multiplex kit: HSTCMAG-28SK-7, Millipore, Billerica, MA, USA) adhering to the manufacturer’s instructions. Data were acquired on a validated and calibrated Luminex 200 system ((Millipore, Billerica, MA, USA). Raw data (mean fluorescence intensities) were captured using the LuminexxPONENT software (v.3.1) and concentrations of immune biomarkers in each sample were interpolated from standard curves using a five-parameter, weighted, logistic regression curve equation in Milliplex Analyst (v.5.1). For each assay, the curve was derived from various concentrations of the cytokine standards assayed in the same manner as test samples. The lower limits of detection (LOD) for specific analytes ranged from 0.12–2.91 pg/mL based on manufacturer specifications. The methodological details including assay method and precision are available at the manufacturer’s website (www.merckmillipore.com).

Serum concentration of High-sensitive C-Reactive Protein (hs-CRP) was measured by performing a particle enhanced immunoturbidimetric assay using an AU680 Beckman coulter as per the manufacturer's instructions (Roche Diagnostics, Indianapolis, IN). The final read-out of the assay was detected as an absorbance change (570 nm), with the magnitude of the change being proportional to the quantity of CRP in the sample. The actual concentration is then determined by interpolation from a calibration curve prepared from calibrators of known concentration. Absolute CRP levels were represented as mg/L. The intra and inter-assay precision was 4.2% and 7.1%, respectively.

Serum MCP-1 levels were measured by AlphaLISA assay (AL509C; Perkin Elmer) as per the manufacturer specifications. The AlphaLISA signal reporting MCP-1 level in the samples was detected at 680nm excitation and 615nm emission using an Enspire multimode reader. A standard curve was generated by plotting the AlphaLISA counts versus the concentration of analyte. Raw counts from the experiment were exported using EnspireManager (version 4.13.3005.1482). The data were analyzed according to a nonlinear regression using the 4-parameter logistic equation and MCP-1 levels were represented as pg/mL. The lower limit of detection of the assay sensitivity was 3.8 pg/mL.

Intestinal alkaline phosphatase (IAP) activity was measured in serum samples with a SensoLytepNPP Alkaline Phosphatase Assay Kit (#71230, AnaSpec, Fremont, CA, USA) according to the recommendations of the manufacturer. AP activity measured in the presence 100 mM L-Phenylalanine (IAP inhibitor) was subtracted from total AP activity to derive levels of serum IAP activity and represented as µg/mg protein.

LBP concentrations were measured in diluted serum samples using the sandwich enzyme-linked immunosorbent assay (ELISA) kit (Human LBP, HK315-02, Hycult Biotech, Uden,The Netherlands), according to the manufacturer's instructions. The standard curve was created by six fold serial dilution of a 50 ng/ml standard solution in duplicate. Measurement of LBP levels was performed at 450nm using EnspireMultomode Plate Reader and the data were exported using EnspireManager and quantified (µg/mL) by standard curve using Graphprism v.6.statistical software. The intra- and inter-assay variability was less than 11.5% and 8.5%, respectively.

**Microbiome sample preparation (DNA extraction from feces)**

Extraction of DNA was performed from 200 mg stool sample from each participant using a standard INRA protocol [3]. After weighing, the samples were chemically lysed by Guanidine thiocyanate and N-Lauryl sarcosine followed by physical lysis which includes the incubation of samples at 70^0^C for one hour. The samples were mechanically lysed by bead beating and the debris, proteins and aromatic compounds were eliminated using PVPP (polyvinylpyrrolidon), RNA removed using RNAse and ethanol used for the precipitation of purified DNA. Finally, DNA was dissolved in 200µl TE Buffer and stored in multiple aliquots at -80^0^C. DNA quality and quantity were measured using Nanodrop. Additionally, the DNA samples were run on a 1% agarose gel electrophoresis for quality checking.

**Sequencing of 16S rRNA genes**

The variable regions (V1-V5) of the 16S rRNA genes were amplified using 27F(C1) and 926R(C5) primersin 50μl reaction volume using 0.1 ng of fecal DNA. We used 5-6 nucleotide long barcodes in the reverse primer to label the amplicon of each sample (Additional File 2). The PCR amplified products (950-bp) were gel purified using QIAquick gel elution kit (Qiagen, Germany). The quality of the DNA library was monitored using the High sensitivity DNA chip compatible to 2100 Bioanalyzer (Agilent, USA). Library quantitation was done using PicoGreen dye in QubitFluorometer (Invitrogen, USA). Sequencing of the equimolar libraries was performed in 454 GS FLX+ pyrosequencer platform (Roche, USA) in two different regions in one pico-titre plate. All the sequencing was done at the Centre for Human Microbial Ecology at the Translational Health Science and Technology Institute. Sequence reads obtained in FASTQ format were evaluated by FASTQC (http://www.bioinformatics.babraham.ac.uk/projects/fastqc/), using default parameters. In addition to samples collected from volunteers recruited in this study, 16S rRNA gene sequencing was also performed for additional microbiome samples (a total of 864 samples) collected from Indian and Danish volunteers with type 2 diabetes for an allied study, using the same protocols and multiplexed sequencing runs described above.

**Microbiome sequence data processing**

A total of 18,380,379 quality filtered (PHRED score >20) reads encompassing the V1-V5 region of the 16S rRNA gene were obtained for the 864 microbiome samples, out of which 11,479,254 reads were subsequently demultiplexed and assigned to 537 samples corresponding to the current study (Indians: 137 NG + 141 PD; Danes: 138 NG + 121 PD) using sequencing barcode information. V3-V5 regions from all the sequenced reads (having variable read-lengths) were subsequently extracted using V-Xtractor 2.0[4], and any read which didn’t encompass the complete V3-V5 region was not considered for further analysis.

A total of 17,030,870 quality checked and trimmed reads pertaining to all 864 samples were considered for the downstream step of OTU picking (average sequencing depth of 19,712 ± 7774SD reads/sample). OTU picking was performed using an ‘open reference OTU picking’ approach as implemented in the QIIME pipe line v1.9.1 [5]. For the process, Greengenes OTUs clustered at 97% identity (Greengenes version 13_8) was used as the reference OTU database[6], while UCLUST v1.2.22q[7]was chosen as the preferred OTU picking method (‘uclust_ref’ run with default parameters for clustering sequences with 97% identity). Representative sequences from each of the OTUs were used for annotating corresponding taxonomic lineages (using the tool dada2[8] considering SILVA database version 132 [9] as a reference). Sparse OTUs containing <0.002 % of the total number of high quality reads sequenced, were removed. A final OTU abundance table with a total of 1897 OTUs, including 1471 OTUs bearing correspondence to OTUs already catalogued in the Greengenes database, as well as 426 de novo OTUs identified from 592 microbiome samples, was considered for downstream analyses. The OTU level abundances were also appropriately cumulated at higher levels of taxonomic hierarchy (e.g. phylum, family, genus etc.) as required for subsequent analyses steps.

OTUs having more than 0.01% abundance in a sample and ubiquitously present in over 80% of samples in a set of samples (e.g. NG or PD), were identified as the ‘core OTUs’ for the considered sample set/cohort. Core OTUs were identified and compared between the Indian and Danish cohorts. Further the core OTUs within the NGT and PD groups for respective geographies were also ascertained. ‘Core genera’ were similarly identified for each of the disease specific and country specific groups, wherein genera having >= 0.1% abundance and >80% ubiquity were identified as the core.

Functional profiles of the bacterial microbiome samples, in terms of KEGG pathways, were imputed from the respective 16S taxonomic profiles, using the software PiCrust v1.1.0 [10]. In addition, the default PiCrust results obtained as 'gene family abundances' in terms of KEGG orthologs (KO) were further curated to depict the relative abundance in terms of KEGG functional modules. For this purpose, a BRITE hierarchy file with the entire list of KEGG functional modules and its association with KO identifiers was downloaded from https://www.genome.jp/kegg/module.html, and parsed using in-house scripts. The eukaryotic pathways/ modules were removed before downstream analyses, following the ‘removal of eukaryotic functions’ strategy implemented in Vikodak[11], another tool for imputing functions profiles.

**Statistical analysis**

Alpha diversity metrics (viz. Shannon diversity, Simpson index and OTU richness) were calculated using R Vegan package v2..5.2[12]. T-tests were performed to assess any significant differences between the alpha diversity parameters of samples belonging to different geographies or health status. It may be mentioned here that the average number of sequenced reads per sample were similar across geographies and glycemic status. For instance, while the Danish normoglycemic (NG) and prediabetic (PD) microbiome datasets had average sequencing depth (demultiplexed high quality reads) of 18372 reads/sample and 18709 reads/sample respectively, the Indian microbiome datasets had average depths of 21140 reads/sample for the NG group and 20936 reads/sample for the PD group. Given similar sequencing depths and the fact that DNA sequencing of all samples were performed collectively in one sequencing center, it is unlikely that the microbiome signatures reported in this work would bear artefacts due to biases in sequencing depths. However, given that sample specific variations in sequencing depth may influence calculation of alpha diversity measures like OTU richness, data for all samples were rarefied to match the sample having minimum sequencing depth (~4500 reads/sample) for this particular step. However, considering the possible downsides of rarefaction while trying to identify differentially abundant bacterial taxa, as suggested by McMurdie and Holmes[13] (Plos Comp Biol., 2014, doi:10.1371/journal.pcbi.1003531), rarefied counts were not used while performing the subsequent analyses.

Differences (if any) between the measured phenotypic traits of subjects belonging to different countries/ health status were evaluated using Wilcoxon test(s). P values were corrected for multiple testing using Benjamini-Hochberg (BH) correction. Country effect (DK vs. IN) on inflammatory biomarkers was analysed by using analysis of covariance (ANCOVA) in a linear mixed model framework with subject Hba1c as covariate (using ‘aov’ function in R). Country effect (adjusting for Hba1c) in terms of average difference in IN with respect to DK was estimated by a post-hoc t-test (R packages multcomp v1.4-8 and effects v4.0 used).

PCoA plots based on taxonomic profiles (relative OTU abundance) of microbiome samples were generated the R Phyloseq package v1.22.3[14], wherein weighted-unifrac was used as the distance metric. Similar PCoA plot was also generated using imputed functional profile of the microbiome samples (KEGG functional modules) wherein Jensen-Shannon divergence (JSD) was used as the distance metric. The extent of variation explained by geography and disease status was tested with PERMANOVA, using ‘adonis2’ function available in the R Vegan package v2.5.2. Dispersion of the country and disease status specific clusters were evaluated using the ‘betadisper’ function available in the R Vegan package v2.5.2.

A negative binomial Wald test using the R package DESeq2 v1.10.1 [15] was performed to identify the taxonomic groups (at all different levels of taxonomic hierarchy), which were differentially abundant in NGT and PD samples (p_adj_ <=0.05, BH corrected) for Indians and Danish cohorts separately. Further, health status specific microbiome signatures were also evaluated after pooling together Indian and Danish cohorts, wherein the negative binomial Wald test results were corrected for geography specific cohort-effect using linear modeling (in DESeq2). Forest-plot based meta-analysis was also performed on the OTUs listed in Table 2, which were observed to be significantly differentially abundant in the pooled analysis (after correcting for geography specific cohort effect). For this purpose, the effect sizes in each geography (i.e. log2fold enrichment of mean abundances in PD with respect to NG in Indian and Danish Cohorts and respective standard errors) were computed using negative binomial Wald tests separately for the Indian and Danish cohorts. These geography specific effects as well as the summary effect, was calculated and plotted using the R package metaviz v 0.3.1.

Additional negative binomial Wald tests were performed (using DESeq2) separately on Indian and Danish subjects to identify discriminating OTUs, while correcting for certain observed covariates of glycaemic status, viz., waist-to-hip ratio, systolic BP, IL6, TNFα, LBP and IAP, which might also influence the microbiome structure. Corrections were also performed for age and gender of the subjects given that the age and gender distribution of normoglycaemic and prediabetic cohorts from the two countries had some variations. Due to missing values of certain covariates, four of the Indian samples, namely MBD081, MBD087, MBD096 and MBD280 were not considered while correcting for above covariates when performing DESeq2 analysis resulting in 274 Indian samples (NG=134, PD=140) and 259 Danish samples (NG=138, PD=121) being considered for this analysis. It may be noted that, during running the negative binomial Wald tests with DESeq, while correcting for the mentioned covariates in the Danish cohort, coefficient vectors for 62 OTUs (out of 1897) did not converge even after 10,000 iterations. However, in case of the Indian cohort, coefficient vectors pertaining to all OTUs, except three, attained convergence during the DESeq2 runs. Spearman correlations between abundances of differentially abundant microbial OTUs (between NGT and PD subjects) and measured phenotypic traits of the subjects were calculated (using R). The correlations were evaluated separately for the Indian and Danish cohorts. The earlier mentioned four Indian samples MBD081, MBD087, MBD096, MBD280, were also removed while computing these correlations between the discriminating taxa and measured phenotypic traits, due to missing values. Heatmaps depicting identified correlations with p<0.05 were generated using the R gplots package v3.0.1. Further the correlation values between each discriminating OTU against different biochemical and inflammatory markers were also corrected for multiple testing using Benjamini-hochberg correction and p_adj_ values listed. The ‘p.adjust’ function available in R - stats package was used for this purpose.

Random forest (RF) classifier(s) were constructed for classifying PD samples based on gut microbiome composition using R Random forest package (v4.6-12). Abundances of all the bacterial OTUs identified in the NG and PD microbiome samples were used as features while building the classifier(s).

A first RF model was generated considering all features and using default parameters, while evaluating the model performance with area under the curve (AUC) of the receiver operating characteristics (ROC) curve and out-of-bag (OOB) error rate. Towards further exploring the possible benefits of feature selection, a second RF model was built using the following procedure. The whole dataset was randomly split (stratified considering proportions of NG and PD classes) into a training set and an independent test set in the ratio 66:34. This ratio of train:test split was decided in view of the following. First, a 34% test corpus being a significant fraction of the whole data set is likely to provide additional confidence while evaluation of model performance. Further 66:34 train:test split should make the results comparable to those obtained with the RF model created without feature selection due to the following reason. Given that while creating a tree for the RF ensemble from a sample size of N, N samples are drawn with replacements constituting roughly 2/3rd of the total samples, approximately 1/3rd of the remaining samples are considered for estimating OOB error[16]. The training procedure involved 10-fold cross-validation with 10 replicates (i.e. a total of 100 tests). Top 10 discriminating features were selected from each cross-validation fold and ranked based on their cumulative importance (using ‘gini score’). To create a final ‘bagged’ model, the ranked features were progressively added (up to a maximum of 100) into the model, according to their cumulative importance, while evaluating the performance of the bagged model on the training set data (in terms of AUC) after addition of every new feature. The ‘area under curve’ (AUC) of the Receiver Operating Characteristic (ROC), was calculated using the R pROC package v1.9.1. A final ‘bagged’ RF model was arrived at which used an optimal number of most discriminating features (OTUs) while providing the best AUC with the training set data. Subsequently the efficiency of the bagged RF model was evaluated using the test set samples.

**R-Session Info for DESeq2 analysis:**

- R version 3.2.5 (2016-04-14) Platform: x86_64-pc-linux-gnu (64-bit), Running under: Ubuntu 18.04.5 LTS
- Locale: LC_CTYPE=en_IN,  LC_NUMERIC=C, LC_TIME=en_IN, LC_COLLATE=en_IN, LC_MONETARY=en_IN, LC_MESSAGES=en_IN, LC_PAPER=en_IN, LC_NAME=C, LC_ADDRESS=C, LC_TELEPHONE=C,  LC_MEASUREMENT=en_IN, LC_IDENTIFICATION=C
- Attached base packages: parallel, stats4, stats, graphics, grDevices, utils, datasets, methods, base
- Other attached packages: DESeq2_1.10.1, RcppArmadillo_0.7.900.2.0, Rcpp_1.0.5,        SummarizedExperiment_1.0.2, Biobase_2.30.0, GenomicRanges_1.22.4, GenomeInfoDb_1.6.3,    IRanges_2.4.8, S4Vectors_0.8.11, BiocGenerics_0.16.1
- Loaded via a namespace (and not attached):  locfit_1.5-9.1, base64_1.1, lattice_0.20-33, digest_0.6.25, R6_2.4.1, futile.options_1.0.1, backports_1.1.10, acepack_1.4.0, RSQLite_2.2.1, ggplot2_3.3.2, pillar_1.4.6, zlibbioc_1.16.0, rlang_0.4.7, rstudioapi_0.11, data.table_1.13.0, annotate_1.48.0, blob_1.2.1, rpart_4.1-10, Matrix_1.2-4, checkmate_2.0.0, splines_3.2.5, BiocParallel_1.4.3, geneplotter_1.48.0, stringr_1.4.0, foreign_0.8-66, htmlwidgets_1.5.2, bit_4.0.4, munsell_0.5.0, xfun_0.18, pkgconfig_2.0.3, htmltools_0.5.0, nnet_7.3-12, tibble_3.0.3, gridExtra_2.3, htmlTable_2.1.0, Hmisc_4.0-1, XML_3.99-0.3, crayon_1.3.4, grid_3.2.5, xtable_1.8-4, gtable_0.3.0, lifecycle_0.2.0, DBI_1.1.0, magrittr_1.5, formatR_1.7, scales_1.1.1, stringi_1.5.3, XVector_0.10.0, genefilter_1.52.1, latticeExtra_0.6-28, futile.logger_1.4.3, ellipsis_0.3.1, vctrs_0.3.4, Formula_1.2-3, lambda.r_1.2.4, RColorBrewer_1.1-2, tools_3.2.5, bit64_4.0.5,  glue_1.4.2, survival_2.40-1, AnnotationDbi_1.32.3, colorspace_1.4-1, cluster_2.0.3, memoise_1.1.0, knitr_1.30

**References**

1. Dantoft TM, Ebstrup JF, Linneberg A, Skovbjerg S, Madsen AL, Mehlsen J, et al. Cohort description: The Danish study of Functional Disorders. Clin Epidemiol. 2017;9:127–39.

2. Johansen NB, Hansen A-LS, Jensen TM, Philipsen A, Rasmussen SS, Jørgensen ME, et al. Protocol for ADDITION-PRO: a longitudinal cohort study of the cardiovascular experience of individuals at high risk for diabetes recruited from Danish primary care. BMC Public Health. 2012;12:1078.

3. Godon JJ, Zumstein E, Dabert P, Habouzit F, Moletta R. Molecular microbial diversity of an anaerobic digestor as determined by small-subunit rDNA sequence analysis. Appl Environ Microbiol. 1997;63:2802–13.

4. Hartmann M, Howes CG, Abarenkov K, Mohn WW, Nilsson RH. V-Xtractor: an open-source, high-throughput software tool to identify and extract hypervariable regions of small subunit (16S/18S) ribosomal RNA gene sequences. J Microbiol Methods. 2010;83:250–3.

5. Caporaso JG, Kuczynski J, Stombaugh J, Bittinger K, Bushman FD, Costello EK, et al. QIIME allows analysis of high-throughput community sequencing data. Nat Methods. 2010;7:335–6.

6. DeSantis TZ, Hugenholtz P, Larsen N, Rojas M, Brodie EL, Keller K, et al. Greengenes, a chimera-checked 16S rRNA gene database and workbench compatible with ARB. Appl Environ Microbiol. 2006;72:5069–72.

7. Edgar RC. Search and clustering orders of magnitude faster than BLAST. Bioinformatics. 2010;26:2460–1.

8. Callahan BJ, McMurdie PJ, Rosen MJ, Han AW, Johnson AJA, Holmes SP. DADA2: High-resolution sample inference from Illumina amplicon data. Nat Methods. 2016;13:581–3.

9. Pruesse E, Quast C, Knittel K, Fuchs BM, Ludwig W, Peplies J, et al. SILVA: a comprehensive online resource for quality checked and aligned ribosomal RNA sequence data compatible with ARB. Nucleic Acids Res. 2007;35:7188–96.

10. Langille MGI, Zaneveld J, Caporaso JG, McDonald D, Knights D, Reyes JA, et al. Predictive functional profiling of microbial communities using 16S rRNA marker gene sequences. Nat Biotechnol. 2013;31:814–21.

11. Nagpal S, Haque MM, Mande SS. Vikodak--A Modular Framework for Inferring Functional Potential of Microbial Communities from 16S Metagenomic Datasets. PLoS ONE. 2016;11:e0148347.

12. Oksanen J, Blanchet FG, Kindt R, Legendre P, Minchin PR, O’hara RB, et al. Package ‘vegan.’ Community ecology package, version. 2013;2.

13. McMurdie PJ, Holmes S. Waste Not, Want Not: Why Rarefying Microbiome Data Is Inadmissible. PLOS Computational Biology. Public Library of Science; 2014;10:e1003531.

14. McMurdie PJ, Holmes S. phyloseq: an R package for reproducible interactive analysis and graphics of microbiome census data. PLoS ONE. 2013;8:e61217.

15. Love MI, Huber W, Anders S. Moderated estimation of fold change and dispersion for RNA-seq data with DESeq2. Genome Biol. 2014;15:550.

16. Janitza S, Hornung R. On the overestimation of random forest’s out-of-bag error. PLOS ONE. 2018;13:e0201904.
