# Supplementary material for: Trans-ethnic gut microbial signatures of prediabetic subjects from India and Denmark
Source: Genome Med. 2021 Mar 3;13:36. doi: 10.1186/s13073-021-00851-9 (PMC7931552; doi:10.1186/s13073-021-00851-9)
Supplement: Supplementary file 3 — Additional file 3: Table S2. Biochemical parameters measured in Danish and Indian cohorts. Comparison of additional biochemical parameters measured in Danish and Indian cohorts. [file 13073_2021_851_MOESM3_ESM.docx]

Table S2: Comparison of additional biochemical parameters measured in Danish and Indian cohorts.

| **Parameter** | **Median (and IQR) value in Danish samples** | |  | **Median (and IQR) value in Indian samples** | |  | **Wilcoxon test p value**  **(after BH correction)** | | | |
| --- | --- | --- | --- | --- | --- | --- | --- | --- | --- | --- |
|  | **NGT** | **PD** |  | **NGT** | **PD** |  | **NGT vs PD (Denmark)** | **NGT vs PD (India)** | **Denmark vs India (NGT)** | **Denmark vs India (PD)** |
| Cholesterol (mmol/L) | 5.5(1.175) | 5.5(1.3) |  | 4.6(1.1) | 4.7(1.1) |  | 6.29E-01 | 5.50E-01 | **1.15E-10** | **2.36E-10** |
| Creatinine (μmol/L) | 69(18) | 72(22) |  | 71(27) | 62(27) |  | 2.30E-01 | 5.50E-01 | **2.35E-02** | **3.65E-05** |
| HDL (mmol/L) | 1.455(0.625) | 1.39(0.52) |  | 1.06(0.31) | 1.01(0.26) |  | 4.25E-01 | 1.10E-01 | **3.31E-16** | **9.02E-17** |
| LDL (mmol/L) | 3.4(1.1) | 3.4(1.3) |  | 3(0.9) | 3.1(1) |  | 8.48E-01 | 5.53E-01 | **1.19E-05** | **5.23E-04** |
| VLDL (mmol/L) | 0.5(0.4) | **0.6**(0.4) |  | 0.5(0.3) | **0.6**(0.4) |  | **3.58E-03** | **1.23E-02** | 2.28E-01 | **3.58E-01** |
|  |  |  |  |  |  |  |  |  |  |  |

Note: The significantly different parameters (Benjamini Hochberg corrected p_adj_<0.05) in the PD group from each cohort are highlighted in bold face fonts.
