# Supplementary material for: Trans-ethnic gut microbial signatures of prediabetic subjects from India and Denmark
Source: Genome Med. 2021 Mar 3;13:36. doi: 10.1186/s13073-021-00851-9 (PMC7931552; doi:10.1186/s13073-021-00851-9)
Supplement: Supplementary file 4 — Additional file 4: Table S3. Country effect on level of inflammatory biomarkers. Country effect on level of inflammatory biomarkers estimated as differences (%) in means (IN relative to DK) derived from linear mixed models adjusted for HbA1c values. Significant effects after BH correction (padj < 0.05) are indicated with asterisks. [file 13073_2021_851_MOESM4_ESM.docx]

**Table S3:** Country effect on level of inflammatory biomarkers

|  | **Effect(%)** | **95% confidence interval** | **p value** | **p_adj_** |
| --- | --- | --- | --- | --- |
| **Higher in Indians** | | | | |
| hsCRP(mg/L)* | 66.04 | 32.1 | 6.08E-05 | 1.34E-04 |
| TNFα(pg/mL)* | 55.29 | 17.83 | 2.13E-09 | 1.17E-08 |
| IL6(pg/mL) | 42.87 | 46 | 6.76E-02 | 9.30E-02 |
| LBP(µg/mL)* | 30.54 | 9.83 | 2.04E-09 | 1.17E-08 |
| IL23(pg/mL) | 12.58 | 35.17 | 4.83E-01 | 4.93E-01 |
| IL1β(pg/mL) | 10.06 | 28.83 | 4.93E-01 | 4.93E-01 |
| IAP(µg/mL) | 6.56 | 14.64 | 3.79E-01 | 4.64E-01 |
| **Higher in Danes** | | | | |
| IL17A(pg/mL)* | -32.86 | 25.11 | 1.04E-02 | 1.91E-02 |
| IL10(pg/mL) | -33.85 | 33.96 | 5.07E-02 | 7.97E-02 |
| MCP1(pg/mL)* | -51.68 | 21.05 | 1.84E-06 | 5.07E-06 |
| IL13(pg/mL)* | -77.27 | 28.37 | 1.31E-07 | 4.79E-07 |

Country effect on level of inflammatory biomarkers estimated as differences (%) in means (IN relative to DK) derived from linear mixed models adjusted for HbA1c values. Significant effects after BH correction (p_adj_ < 0.05) are indicated with asterisks.
