# Supplementary material for: Trans-ethnic gut microbial signatures of prediabetic subjects from India and Denmark
Source: Genome Med. 2021 Mar 3;13:36. doi: 10.1186/s13073-021-00851-9 (PMC7931552; doi:10.1186/s13073-021-00851-9)
Supplement: Supplementary file 6 — Additional file 6: Figures S1-S6. PDF file with all supplementary figures (Figures S1-S6) with corresponding figure legends. [file 13073_2021_851_MOESM6_ESM.pdf]

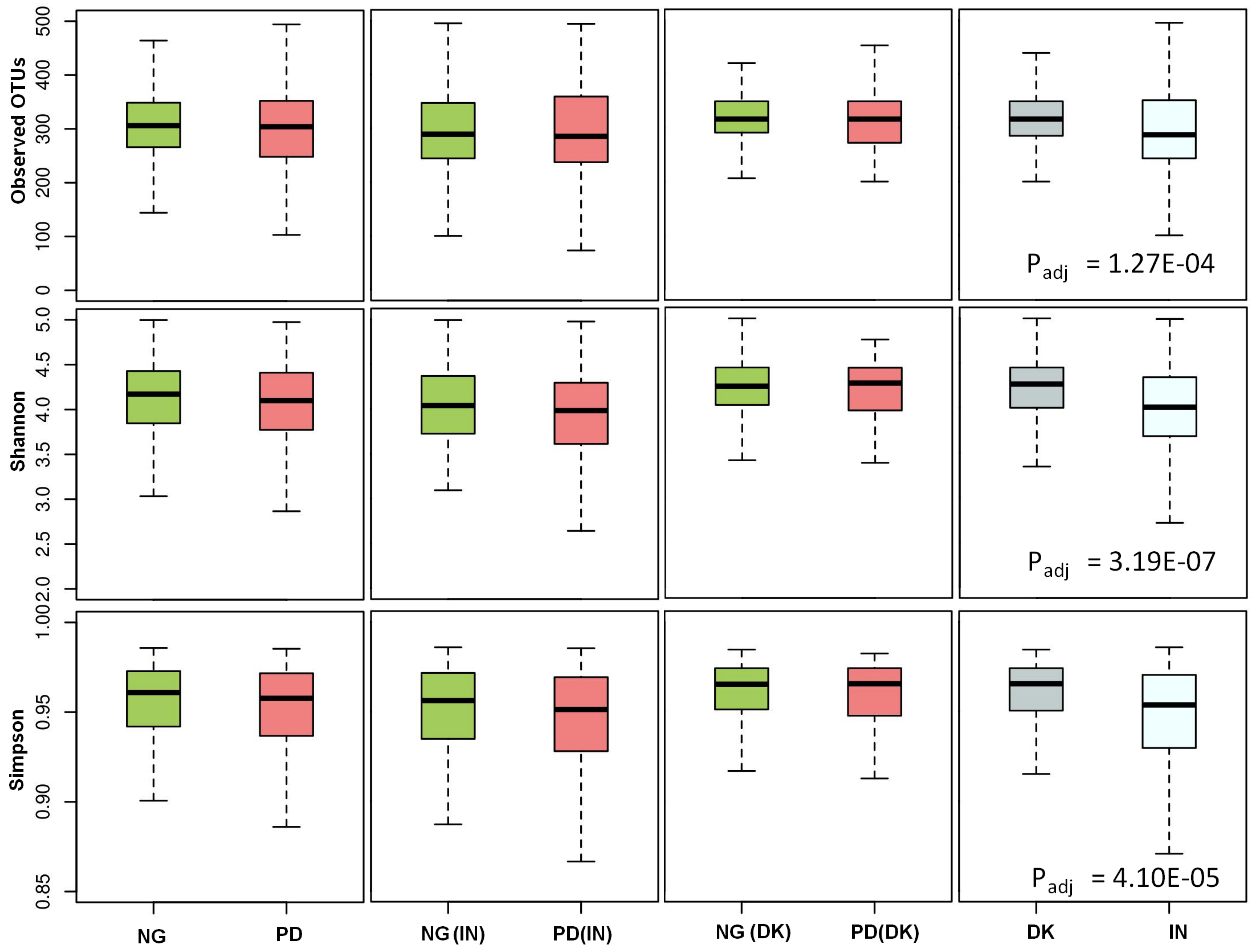

Fig. S1: Alpha-diversity comparison for different cohort groups. Diversity indices calculated for different cohort groups. Any significant differences ($p<0.05$) in diversity have been assessed with t-test, and are indicated in the plots. A taxonomic abundance table rarefied to 4500 reads/sample was used for the calculations. [key -NG: all (IN+DK) samples with normal glucose tolerance; PD: all (IN+DK) samples with pre-diabetes; DK: All Danish samples irrespective of their disease status; IN: All Indian samples irrespective of their disease status]

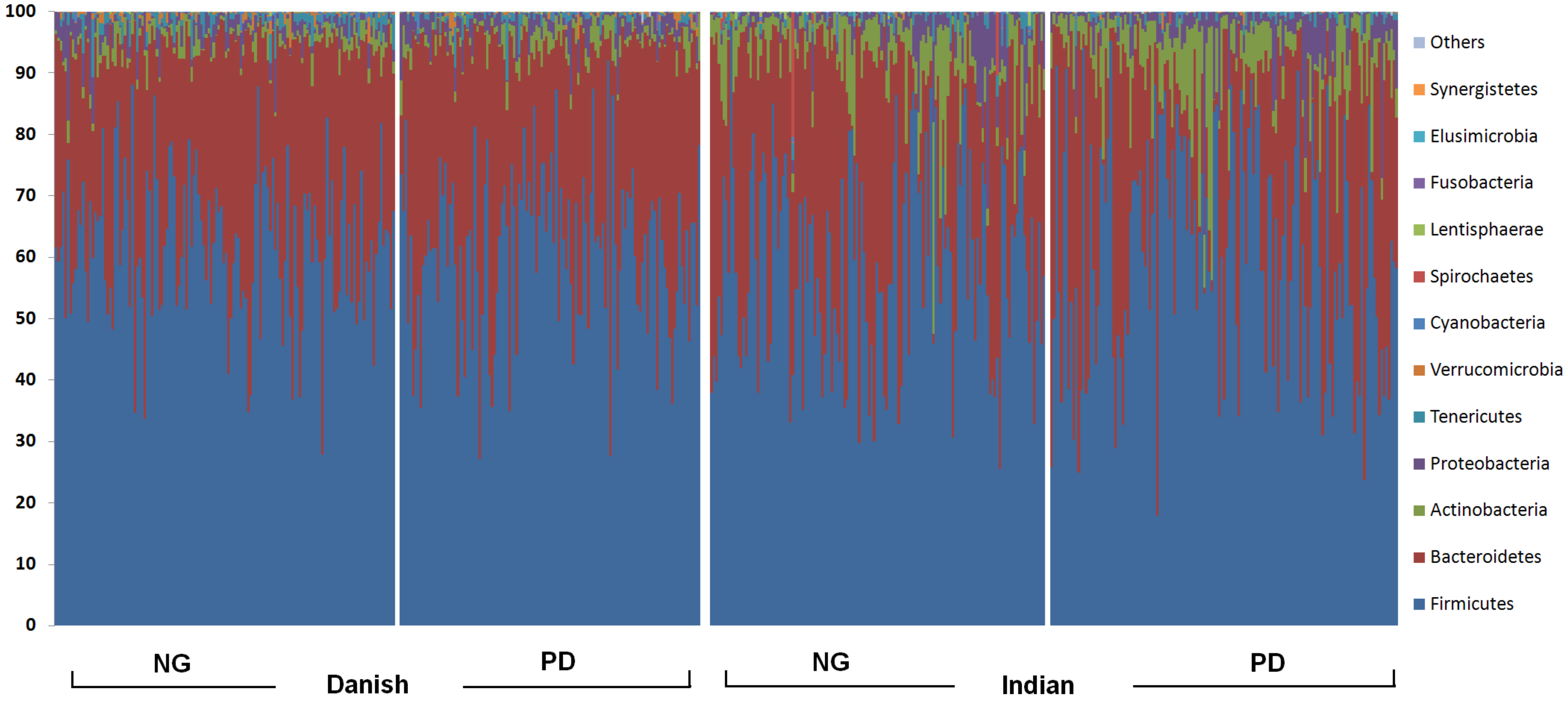

Fig. S2: Taxonomic abundance (percent normalized) at Phylum level for all samples. Taxonomic abundance (percent normalized) at Phylum level for all (537) samples. The 'Others' group reflects the proportion of sequenced reads which could not be assigned at Phylum level.

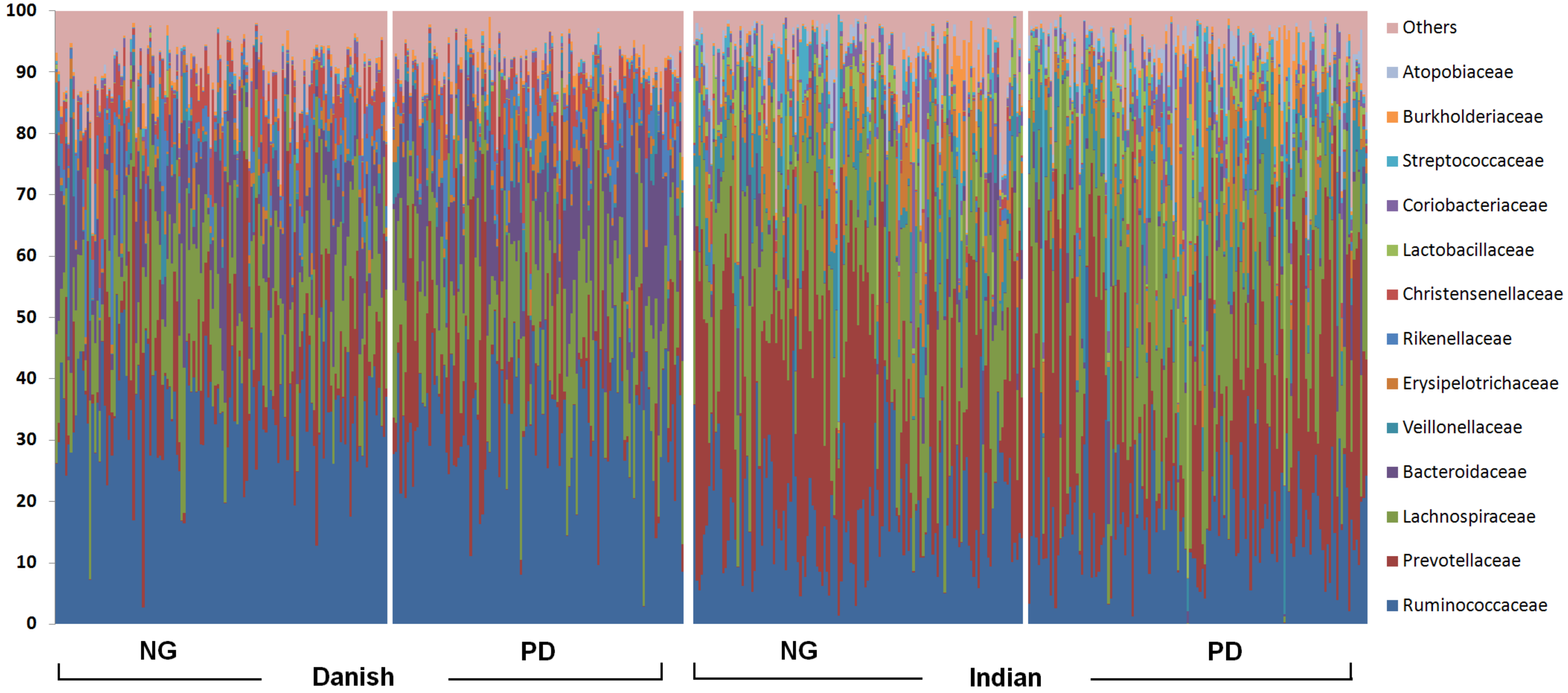

Fig. S3: Taxonomic abundance (percent normalized) at Family level for all samples. Taxonomic abundance (percent normalized) at Family level for all (537) samples. Low abundant families (<1.0% abundance) have been cumulated in the 'Others' group along with the proportion of sequenced reads which could not be assigned at Family level.

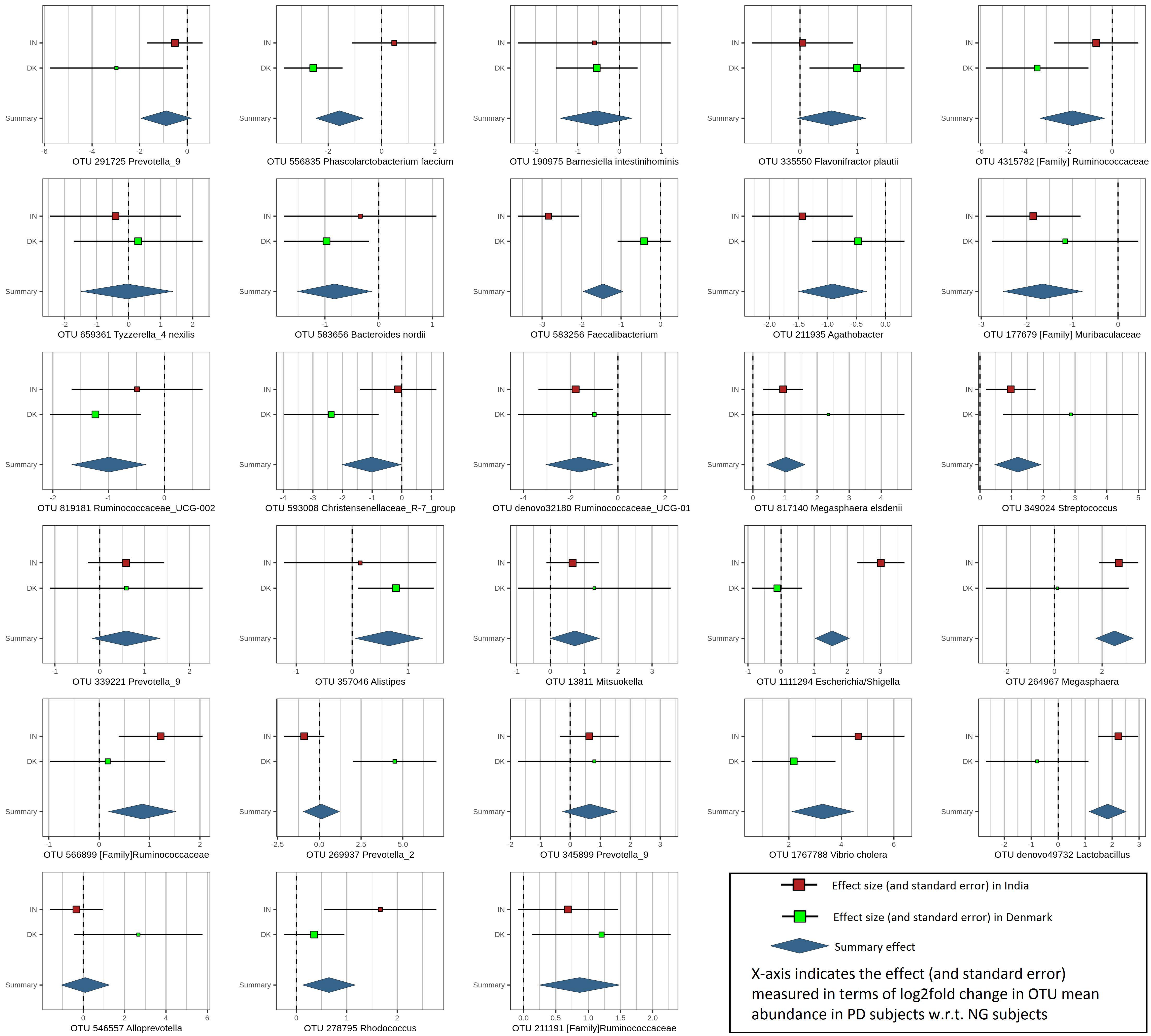

Fig. S4: Meta analysis of differentially identified OTUs in PD and NG subjects from India and Denmark. Forest plot depicting country specific effect size and direction measured in terms of log2fold change in OTU abundance in PD subjects w.r.t. NG subjects, and summary effect considering both geographies. Fold change (and standard error) were computed using the negative binomial Wald test separately for Indian and Danish cohorts.

A

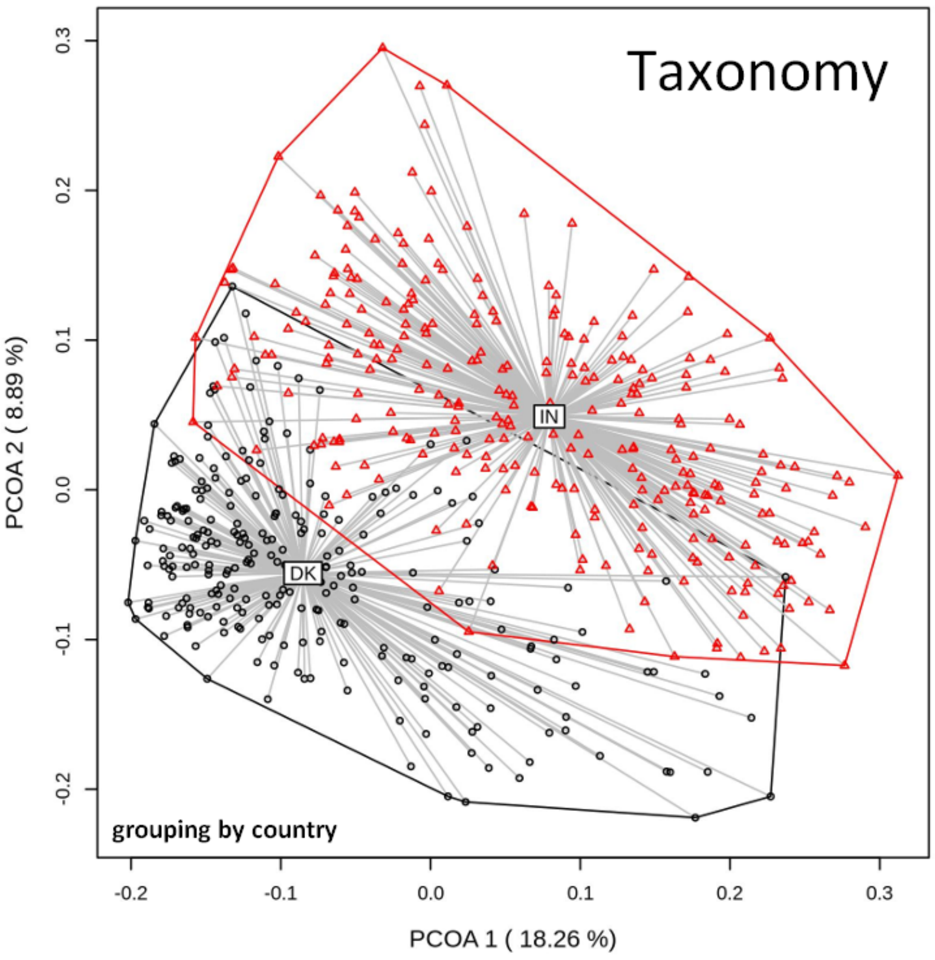

	Df	Sum Sq	Mean Sq	F value	Pr(>F)
Groups	1	0	0	0.18	6.68e-01
Residuals	535	1.85	0	NA	NA

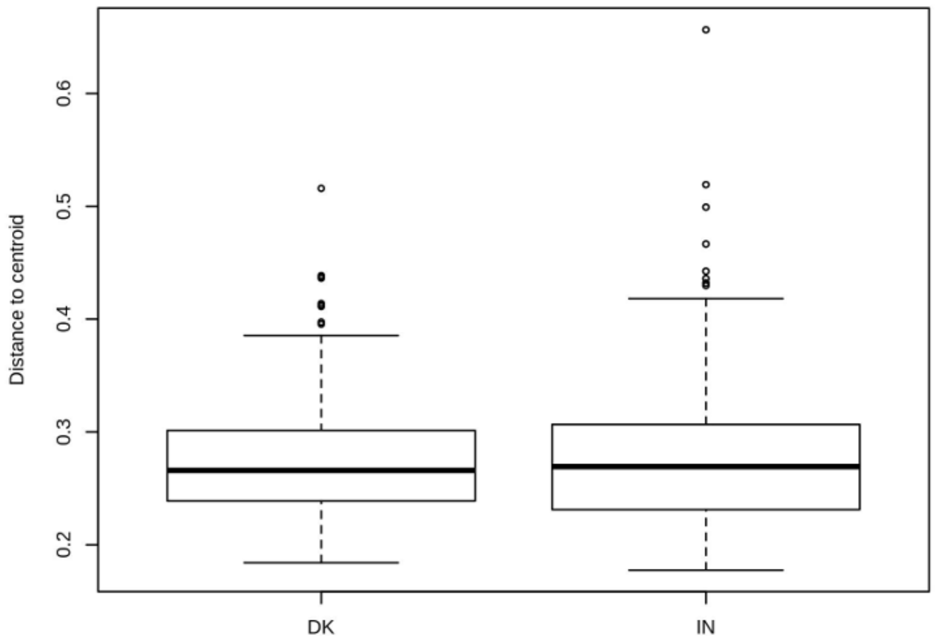

B

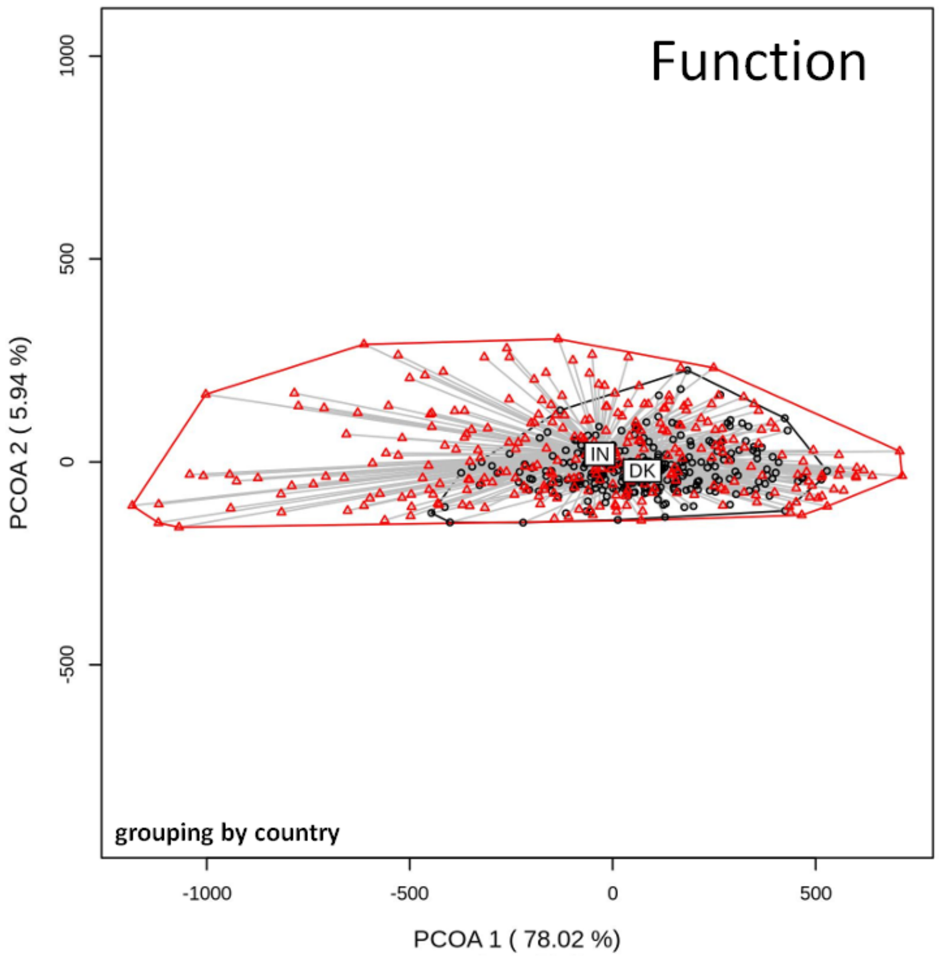

	Df	Sum Sq	Mean Sq	F value	Pr(>F)
Groups	1	3943864.87	3943864.87	129.53	5.03e-27
Residuals	535	16289461.67	30447.59	NA	NA

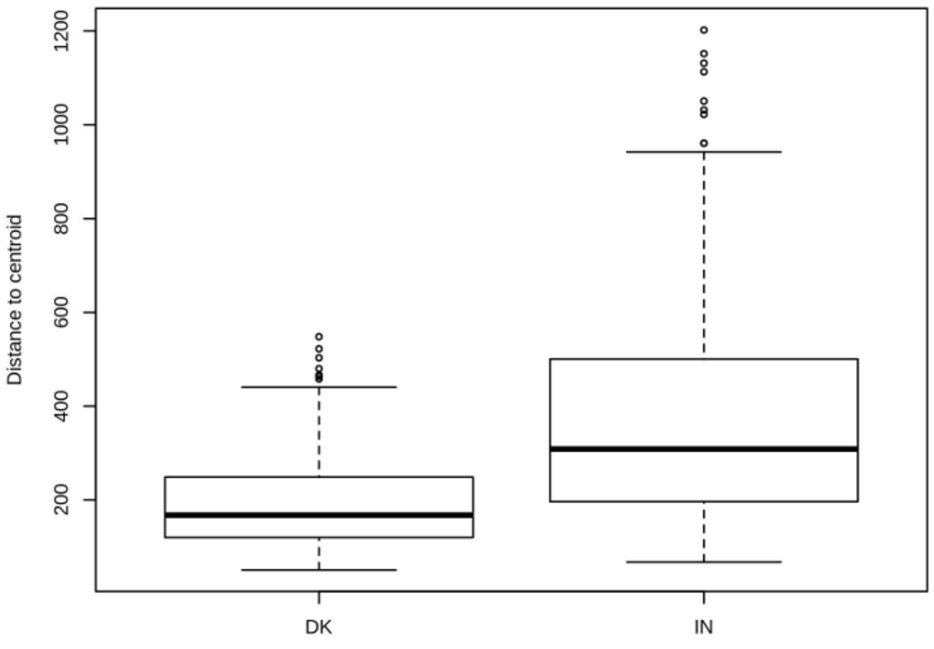

Fig. S5: PCoA based Dispersion patterns of microbiome. Dispersion patterns of microbiome samples according to country of origin (IN or DK). PCoA plots base on - (A) OTU presence using weighted unifrac distances and (B) KEGG functional modules present in different microbiome samples (as inferred with PiCrust) using JSD distances. The microbiome samples have been plotted along the first two principal components. Dispersion of clusters (tested using the 'betadisper' function available in R Vegan package) along with distances of individual microbiome samples from group centroids are also indicated.

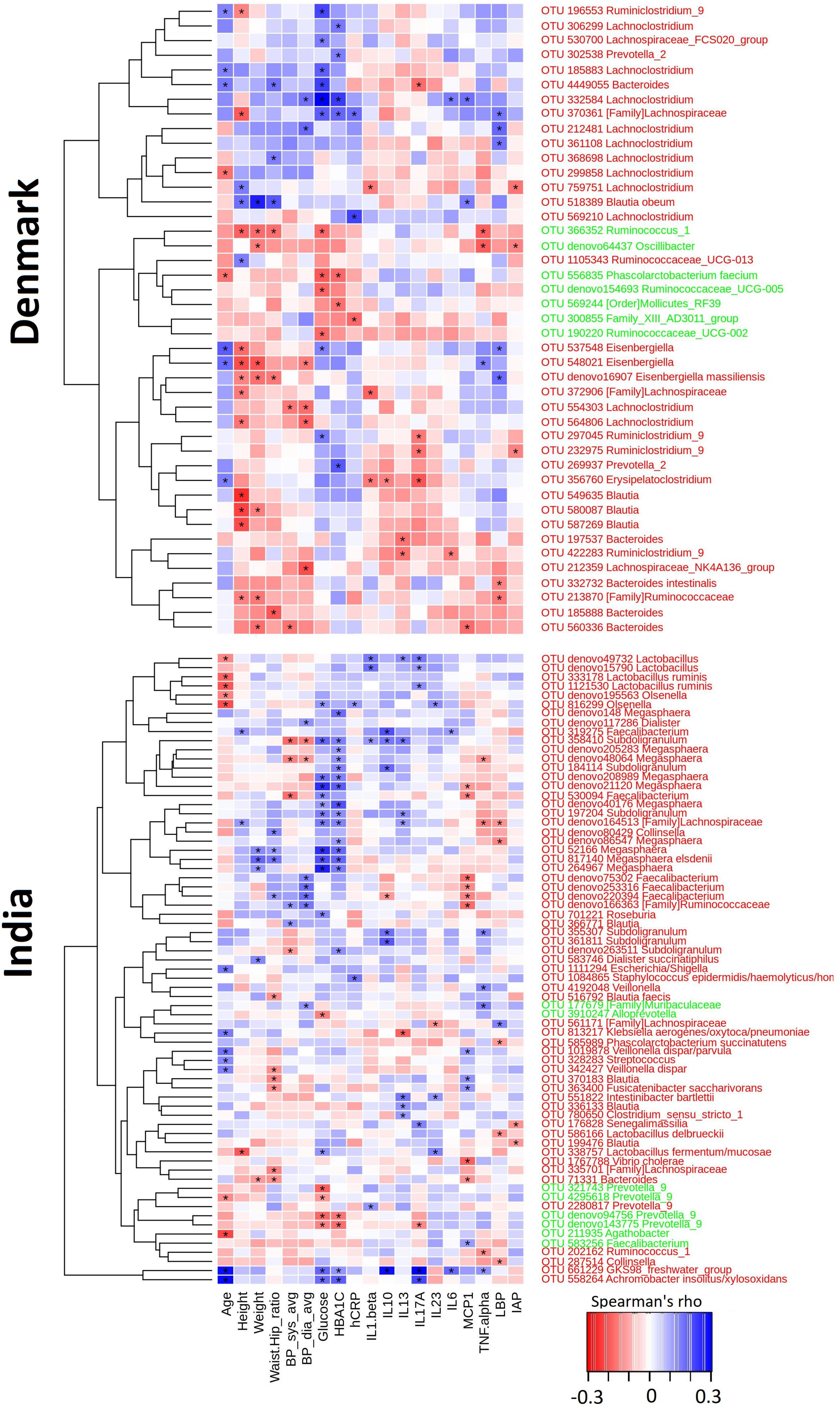

Fig. S6: Correlation between OTU abundances and phenotypic traits.Heatmaps representing correlation between OTU abundances and phenotypic traits of subjects belonging to the Danish and Indian cohorts. The OTUs enriched in NG and PD samples in corresponding geographies are highlighted in green and red respectively. Significant correlations (p <0.05) are indicated in the heatmap with an asterisk (*) symbol.
