# Supplementary material for: Trans-ethnic gut microbial signatures of prediabetic subjects from India and Denmark
Source: Genome Med. 2021 Mar 3;13:36. doi: 10.1186/s13073-021-00851-9 (PMC7931552; doi:10.1186/s13073-021-00851-9)
Supplement: Supplementary file 8 — Additional file 8: Table S6. Core OTUs identified in Indian and Danish cohorts (ignoring disease status). OTUs which are present in at least 80% of the samples belonging to a particular cohort, having a minimum (normalized) abundance of 0.01%, have been defined to constitute the core. [file 13073_2021_851_MOESM8_ESM.docx]

Table S6: Core OTUs identified in Indian and Danish cohorts (ignoring disease status). OTUs which are present in at least 80% of the samples belonging to a particular cohort, having a minimum (normalized) abundance of 0.01%, have been defined to constitute the core.

| **OTU IDs** | **Taxonomic Affiliation** |  | **OTU IDs** | **Taxonomic Affiliation** |
| --- | --- | --- | --- | --- |
|  |  |  |  | |
| **Core OTUs in Indian + Danish cohorts** | |  | **Core OTUs in Danish cohort** | |
| 365181 | *Collinsella aerofaciens* ^#^ |  | 3579707 | *Odoribacter splanchnicus* ^+^ |
| 368175 | *Collinsella aerofaciens* ^#^ |  | 183480 | *Alistipes putredinis* ^+^ |
| 370183 | *Blautia* * |  | 583117 | *Bacteroides thetaiotaomicron* ^+^ |
| 516792 | *Blautia faecis* * |  | 589277 | *Bacteroides dorei/vulgatus* ^+^ |
| 518389 | *Blautia obeum* * |  | 535375 | *Bacteroides fragilis/ovatus* ^+^ |
| 364824 | *Blautia obeum* * |  | 584541 | *Bacteroides uniformis* ^+^ |
| 1076587 | *Dorea formicigenerans* * |  | 589071 | *Bacteroides* ^+^ |
| 369486 | *Lachnospira pectinoschiza* * |  | 578016 | *Parabacteroides* ^+^ |
| 358104 | *Lachnoclostridium* * |  | 585914 | *Parabacteroides distasonis* ^+^ |
| 362767 | *Fusicatenibacter* * |  | 357046 | *Alistipes finegoldii/onderdonkii* ^+^ |
| 363400 | *Fusicatenibacter saccharivorans* * |  | 772282 | *Alistipes* ^+^ |
| 364092 | *Agathobacter* * |  | 1105343 | *Ruminococcaceae_UCG-013* * |
| 369227 | *Dorea longicatena* * |  | 346302 | *Lachnospira* * |
| 518438 | *Agathobacter* * |  | 212481 | *Lachnoclostridium* * |
| 536910 | *Lachnoclostridium* * |  | 353292 | *Lachnospiraceae_NK4A136_group* * |
| 708680 | *Roseburia faecis* * |  | 360329 | *Lachnospiraceae_NK4A136_group* * |
| 345542 | *Roseburia inulinivorans* * |  | 524404 | [Family]Lachnospiraceae * |
| 528362 | *Roseburia* * |  | 1040889 | *Lachnospiraceae_NK4A136_group* * |
| 325437 | *Ruminococcaceae_UCG-002 bacterium* * |  | 194374 | *Lachnoclostridium* * |
| 369429 | [Family]Lachnospiraceae * |  | 373928 | *Christensenellaceae_R-7_group* * |
| 470382 | [Family]Lachnospiraceae * |  | 366633 | *Ruminococcaceae_UCG-005* * |
| 509416 | *Anaerostipes hadrus* * |  | 327818 | *Ruminococcaceae_UCG-002* * |
| 591671 | *Coprococcus_3 comes* * |  | 766113 | *Ruminococcaceae_UCG-005* * |
| 520774 | *Faecalibacterium prausnitzii* * |  | 335097 | *Ruminococcaceae_UCG-003* * |
| 528652 | *Faecalibacterium CM04-06* * |  | 353155 | *Ruminococcaceae_UCG-002* * |
| 529940 | *Faecalibacterium prausnitzii* * |  | 359563 | *Ruminococcaceae_UCG-005* * |
| 851865 | *Faecalibacterium cf./prausnitzii* * |  | 585227 | *Oscillibacter* * |
| 1062061 | *Ruminococcaceae_UCG-002* * |  | 340170 | *Ruminiclostridium_5* * |
| 355307 | *Subdoligranulum* * |  | 580008 | *Erysipelotrichaceae_UCG-003* * |
| 361811 | *Subdoligranulum* * |  |  |  |
| 367213 | *Subdoligranulum* * |  | **Core OTUs in Indian cohort** | |
| 551902 | *Butyricicoccus* * |  | 183104 | *Senegalimassilia* ^#^ |
|  |  |  | 530653 | *Prevotella_9* ^+^ |
|  |  |  | 568118 | *Prevotella_9* ^+^ |
|  |  |  | 588929 | *Prevotella_9* ^+^ |
|  |  |  | 1121530 | *Lactobacillus ruminis* * |
|  |  |  | 941096 | *Streptococcus* * |
|  |  |  | 780650 | *Clostridium_sensu_stricto_1* * |
|  |  |  | 199753 | *Dorea* * |
|  |  |  | 366771 | *Blautia* * |
|  |  |  | denovo148146 | *Blautia* * |
|  |  |  | 752354 | *Coprococcus_1 catus* * |
|  |  |  | 185594 | *Marvinbryantia* * |
| Key: ^#^ Actinobacteria  ^#^ Actinobacteria | |  | 336375 | [Family]Lachnospiraceae * |
| ^+^ Bacteroidetes  ^+^ Bacteroidetes | |  | 212634 | [Order]Clostridiales * |
| * Firrmicutes  * Firrmicutes | |  | 365484 | [Order]Clostridiales * |
|  |  |  | 817140 | *Megasphaera elsdenii* * |
